# Permanent magnetic droplet–derived microrobots

**DOI:** 10.1126/sciadv.adw3172

**Published:** 2025-07-09

**Authors:** Yuanxiong Cao, Ruoxiao Xie, Philipp W. A. Schönhöfer, Ross Burdis, Richard Wang, Rujie Sun, Kai Xie, Jiawen Zou, Xin Song, Qiao You Lau, Junliang Lin, Jang Ah Kim, Dimitar Georgiev, Jiyuan Tang, Ho-Cheung Ng, Olga Bibikova, Yuyang Zuo, Xiangrong L. Lu, Sharon C. Glotzer, Molly M. Stevens

**Affiliations:** ^1^ Department of Physiology, Anatomy and Genetics, Department of Engineering Science, Kavli Institute for Nanoscience Discovery, University of Oxford, South Parks Road, Oxford OX1 3QU, UK.; ^2^ Department of Materials, Department of Bioengineering and Institute of Biomedical Engineering, Imperial College London, London SW7 2AZ, UK.; ^3^ Department of Chemical Engineering, University of Michigan, Ann Arbor, MI 48109, USA.; ^4^ Department of Computing and UKRI Centre for Doctoral Training in AI for Healthcare, Imperial College London, London SW7 2AZ, UK.; ^5^ Biointerfaces Institute, University of Michigan, Ann Arbor, MI 48109, USA.

## Abstract

Microrobots hold substantial potential for precision medicine. However, challenges remain in balancing multifunctional cargo loading with efficient locomotion and in predicting behavior in complex biological environments. Here, we present permanent magnetic droplet–derived microrobots (PMDMs) with superior cargo loading capacity and dynamic locomotion capabilities. Produced rapidly via cascade tubing microfluidics, PMDMs can self-assemble, disassemble, and reassemble into chains that autonomously switch among four locomotion modes—walking, crawling, swinging, and lateral movement. Their reconfigurable design allows navigation through complex and constrained biomimetic environments, including obstacle negotiation and stair climbing with record speed at the submillimeter scale. We also developed a molecular dynamics–based computational platform that predicts PMDM assembly and motion. PMDMs demonstrated precise, programmable cargo delivery (e.g., drugs and cells) with postdelivery retrieval. These results establish a physical and in silico foundation for future microrobot design and represent a key step toward clinical translation.

## Teaser

Permanent magnetic droplet–derived microrobots have enhanced cargo loading and locomotion capabilities.

## INTRODUCTION

Modular microrobots with reconfigurability can adapt to environmental variations through versatile locomotion modes for targeted medical treatments (*1*–*5*). Magnetic modular microrobots that can be remotely controlled by magnetic fields are particularly suitable for minimally invasive delivery of therapeutic payloads deep into damaged or diseased tissues (*6*–*11*). However, challenges persist in improving cargo loading capacity within microrobots without compromising their locomotion efficiency.

Responsive hydrogels, which deform in response to external stimuli, can adapt to complex in vivo environments and offer great potential for therapeutic delivery because of their hydrated nature and tunable biochemical properties (*12*–*17*). Yet, forces triggering hydrogel deformations are typically insufficient to generate effective movement (*18*, *19*). To address this issue, one strategy involves incorporating soft-magnetic components within the hydrogel network to enhance locomotion. Recent work demonstrated that embedding soft-magnetic components within soft beads enables the generation of flexible magnetic assemblies (*20*–*22*), which is a promising strategy to balance mechanical adaptability and functional responsiveness under magnetic control. However, soft-magnetic components are susceptible to demagnetization, which reduces their deformation capability and weakens the connectivity (*23*, *24*), leading to structural collapse in dynamic environments (*25*–*27*). Hard-magnetic components, e.g., neodymium-iron-boron (NdFeB), with high coercivity and permanent magnetization, can retain their transformed structures across a range of magnetic fields and even without an external field (*8*, *28*, *29*). However, their high density can cause aggregation and fragility when integrated into low-viscosity materials like hydrogels (*27*). Traditionally, hard-magnetic components have been mainly used in rigid polymeric matrices, forming bulky structures with limited cargo release capabilities (*8*, *30*, *31*).

To address these challenges, we synthesized permanent magnetic hydrogel–based particles, namely permanent magnetic droplet–derived microrobots (PMDMs), to enhance cargo loading capacity while maintaining multimodal locomotion abilities. We produced PMDMs in a Janus configuration comprising a hydrogel phase and a hard-magnetic phase using a cascade tubing microfluidic device that allows rapid mass production. The hydrogel phase has exceptional cargo loading performance, capable of carrying nanoparticle drugs and therapeutic cells. The hard-magnetic phase enables self-assembly into PMDM chains that exhibit multimodal locomotion behaviors, including crawling, walking, swinging, and lateral movement, via magnetic control. The PMDM chains can also reconfigure to navigate confined spaces by disassembling into shorter-chain fragments and to overcome tall obstacles by merging into elongated chains. We also successfully developed a computational model based on molecular dynamics (MD) simulations and informed by finite element (FE) calculations to get further insight into the dynamic self-assembly and disassembly, multimodal locomotion, and collective behavior of PMDMs. We then validated the applicability of PMDMs in clinically relevant scenarios: endoscope-assisted delivery to a 3D-printed human cartilage model and a porcine intestine model. The highly controlled locomotion and dynamic cargo loading highlight the great potential of PMDMs for enabling precise medical treatment and accelerating the clinical translation of microrobotic therapeutic delivery platforms.

## RESULTS

### Creation of cascade tubing microfluidics–engineered PMDMs

**Fig. 1.**
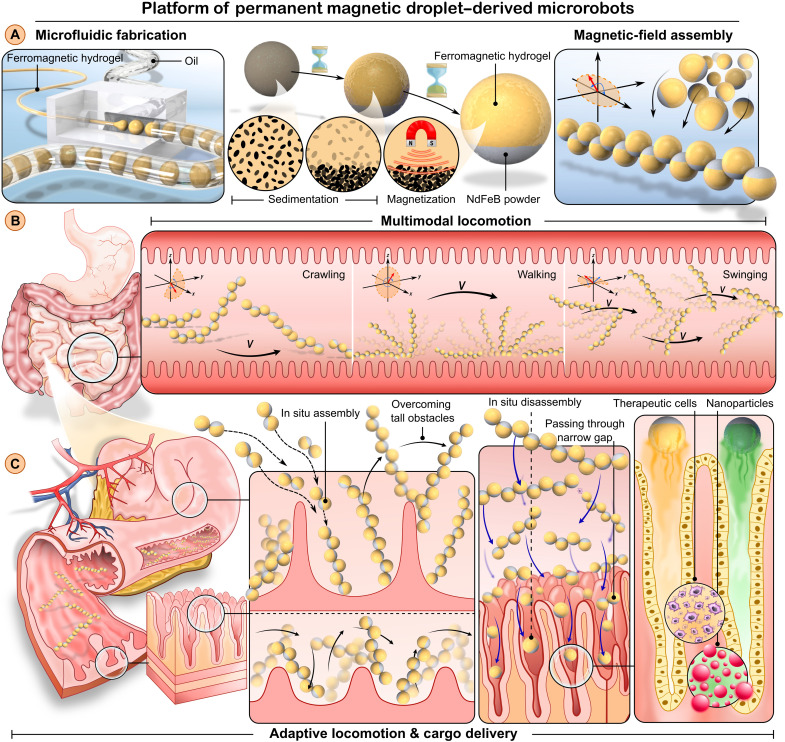
Fabrication, multimodal and adaptive locomotion, and delivery of PMDMs. (**A**) PMDMs were fabricated using cascade tubing microfluidics, where the ferromagnetic hydrogel was pinched off periodically under the oil shearing effect, forming monodispersed ferromagnetic droplets. The gravitational sedimentation of ferromagnetic microparticles (NdFeB, 5 μm) leads to a distinct separation between the magnetic and hydrogel phases, yielding Janus microrobots. (**B**) Multimodal locomotion of assembled PMDMs was programmatically triggered by alternating magnetic fields, including crawling, walking, and swinging. (**C**) Adaptive locomotion and multifunctional delivery of PMDMs. The PMDM chains loaded with cells or drugs can assemble to overcome tall obstacles and disassemble to cross narrow channels. Figure produced using SolidWorks and Inkscape.

The PMDMs were generated using cascade tubing microfluidics (Fig. 1A, left, and movie S1), which enabled rapid and high-throughput manufacture of ferromagnetic droplets at the rate of 300 droplets per minute. In this process, the ferromagnetic composite hydrogel precursor, consisting of biocompatible hydrogels and NdFeB microparticles (around 5 μm), was introduced into a microfluidics device where it forms a continuous thread. Concurrently, a stream of oil applies shear force on the precursor fluid, causing the thread to break into monodisperse ferromagnetic droplets. The presence of NdFeB microparticles within the hydrogel matrix induced a distinct separation between the ferromagnetic and hydrogel phases because of gravitational sedimentation, resulting in Janus droplets (Fig. 1A, middle, fig. S1, and movie S1). The Janus ferromagnetic droplets were subsequently polymerized and magnetized to form PMDMs. The ferromagnetic phase enabled controllable assembly into PMDM chains (Fig. 1A, right), as well as multimodal and adaptive motion (Fig. 1B). The PMDM nonmagnetic hydrogel phase was fabricated using both rigid polyethylene glycol diacrylate (PEGDA) and soft gelatin/alginate hydrogels, which provided excellent cargo delivery abilities. We also investigated the use of PMDMs to deliver multiple cargoes, including nanoparticle drugs and therapeutic cells, in intricate biological environments with reconfiguration and tunable release rate (Fig. 1C).

### Characterization of PMDMs

**Fig. 2.**
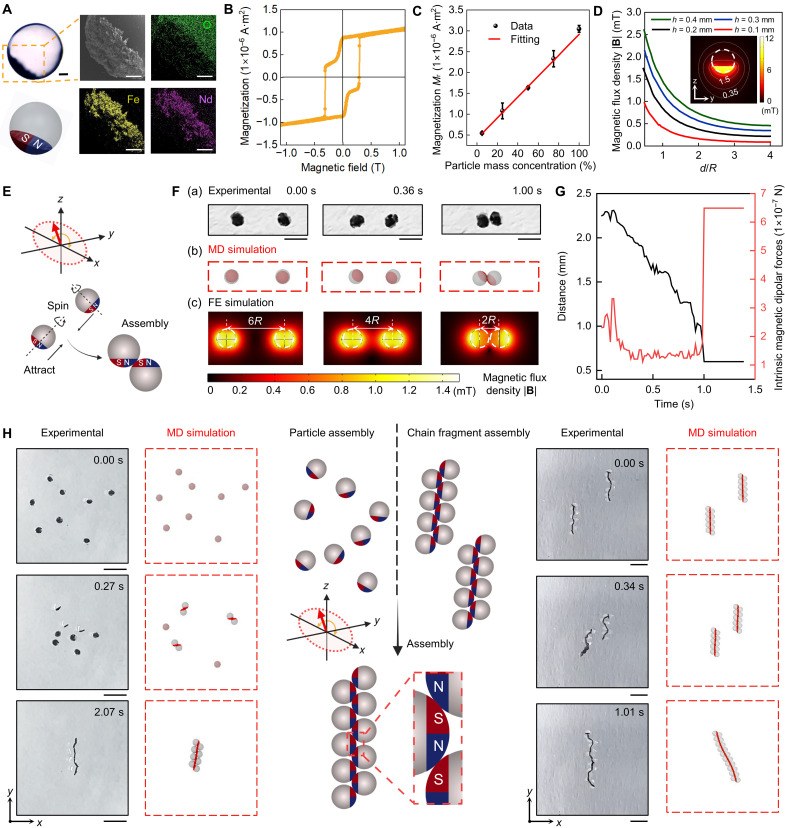
Characterization and self-assembly of PMDMs. (**A**) Optical microscopy and EDS elemental mapping of a microrobot. Scale bars, 100 μm. (**B**) Magnetic hysteresis loop of a single microrobot [comprising an 80% (v/v) PEGDA matrix with 25% (w/v) NdFeB microparticles] measured with a vibrating sample magnetometer. (**C**) Linear dependence of the remnant magnetization (*M*_r_) on the mass concentration of embedded magnetic particles. All values are represented as the means ± SD (*n* = 3). (**D**) Magnetic flux density **B** along the central axis of PMDMs plotted as a function of the normalized working distance *d*/*R* for varying sedimentation heights *h* (*h* = 0.1, 0.2, 0.3, and 0.4 mm). *R* represents the PMDM radius, and *d* indicates the distance to the center of a PMDM. The inserted image is the FE-simulated magnetic field distribution surrounding a single PMDM with *h* = 0.2 mm. (**E**) PMDM dimers spin around a precession axis *z*, resulting in their eventual assembly under a horizontal oscillating magnetic field. (**F**) Time-lapse images showing two approaching PMDMs at specific time intervals, shown as experimental data (a), MD simulation (b), and FE simulation (c). Scale bars, 1 mm. (**G**) Quantitative analysis of center-to-center distance *D* and the corresponding intrinsic magnetic dipolar forces as PMDMs approach each other. Intrinsic magnetic dipolar forces are derived from simulation analyses. (**H**) Assembly of individual PMDMs (left) and PMDM chain fragments (right) under a horizontal oscillating magnetic field, shown as experimental and MD simulation images. Scale bars, 2 mm. Created in BioRender. Cao, Y. (2025) https://BioRender.com/n2tq29v.

Having established a robust high-throughput fabrication method, we next carried out a comprehensive and multimodal characterization of PEGDA-based PMDMs. The interface between the ferromagnetic and hydrogel phases is clearly visible (Fig. 2A). Energy-dispersive x-ray spectroscopy (EDS) analysis further confirmed the settling of the NdFeB microparticles at the bottom of the PMDM (Fig. 2A).

The PMDM diameter *D* was tailored by adjusting the size of the collection tubing. For all tubing sizes, there is a normal *D* distribution with limited variation (fig. S2). Collection tubing with sizes of 200, 500, and 1000 μm resulted in the formation of PDMDs with mean diameters of 325 ± 13, 575 ± 38, and 1082 ± 36 μm, respectively. Given the cargo loading capacity and injectability, 500-μm tubing was used for all other experiments, unless otherwise specified (fig. S2). The sedimentation height *h* of the PMDMs was controlled by adjusting the mass concentration of the embedded NdFeB microparticles (fig. S3 and section S1), where higher concentrations resulted in a greater *h*. The magnetization hysteresis curve reflects typical hard-magnetic behavior with a remanent magnetization intensity *M*_r_ of 0.87 × 10^−6^ A·m^2^ and a high coercivity *H*_c_ of 300 mT (Fig. 2B). The *M*_r_ correlates linearly with the mass concentration of the integrated NdFeB microparticles (Fig. 2C and fig. S4), where a higher concentration of NdFeB microparticles led to higher *M*_r_. FE simulation reveals that PMDMs display elliptical magnetic field contour lines in the *xz* plane, which are symmetrical along the magnetization direction but asymmetrical in the nonmagnetization direction (Fig. 2D and fig. S5A). Notably, a higher *h* corresponds to a stronger magnetization field distribution. For instance, a microrobot with *h* = 0.4 mm has a magnetization field twice as strong as that of *h* = 0.1 mm at the same distance (Fig. 2D). The magnetization distribution of the PMDMs decays exponentially as a function of the distance *d* to the centroid of the PMDMs ( $\propto 1/d^{3}$ ), with a rapid decline in magnetic strength at *d* = 2*R* (where *R* is the radius of the PMDM) and a more gradual decrease at *d* > 2*R* (Fig. 2D).

### Oscillation-induced dynamic self-assembly into PMDM chains

To study the dynamic assembly of PMDM pairs into dimers, we exposed two PMDMs to a precessing magnetic field [Fig. 2, E and F (a), fig. S6, and movie S2], which is known to induce synchronized motion (*32*). In the beginning, at a distance 6*R*, the PMDMs stood upright with their heavier magnetic side at the bottom and experienced magnetic torques, prompting synchronized oscillation [Fig. 2F (a)]. Because of dipole-dipole interactions, PMDMs moved closer together with their magnetic coupling intensifying while approaching.

To further characterize the assembly process, we developed a particle-based model for PMDMs based on MD simulation. Each PMDM is described as a spherical particle containing a magnetic cap with sedimentation height *h* (fig. S7). The magnetic cap was assigned a permanent magnetic dipole perpendicular to the direction of its symmetry axis (see Materials and Methods for details). MD simulation fully reproduced the assembly process, closely matching the experimental timeline [Fig. 2F (b) and movie S2]. FE simulation shows the concentration of the magnetic flux density between the particles as they approach one another, with closer distances resulting in stronger interactions [Fig. 2F (c)]. When the particles came in contact, the intrinsic magnetic dipolar forces between the two particles were strong enough to flip the magnetic parts toward each other, causing them to dimerize into a stable, balanced configuration with a slight offset as depicted in Fig. 2G. MD simulation also predicted the same dynamic assembly behavior into dimers for different *h* values (1/10 D < *h <* 9/10 D) (fig. S8). We found that at lower *h* (indicating fewer NdFeB microparticles), the dipole forces were too weak to flip the PMDMs and induce dimer formation, while at very high *h*, the magnetic part of the PMDMs was too heavy for the microrobots to flip. At intermediate *h*, we were able to dictate and increase the offset angle with increasing *h*, as verified experimentally.

Next, we explored experimentally and computationally the self-assembly of PMDMs into chains (number of PMDMs per chain is *N*_s_ = 8). When subjected to a precessing magnetic field, the microrobots approached each other. Here, they rapidly assembled first into dimers and trimers before they formed a single PMDM chain of eight PMDMs where the dipole moments of each PMDM aligned (Fig. 2H, left). Because of the offset angle, already observed in the dimerization process, the particles arranged in a zigzag formation within the chain. Moreover, the efficiency (percentage of the microrobot population incorporated into a single chain) of the PMDM chain assembly depends on *h*. A lower *h* implies a limited ferromagnetic phase area and weaker magnetic force, which resulted in a looser connection of the PMDMs (fig. S9 and movie S3), making it challenging to form a cohesive chain and instead remaining as fragments. In contrast, a larger *h* induces a stronger magnetic force, facilitating a stronger association during assembly (fig. S10). PMDMs with varying sizes can all be assembled into PMDM chains (fig. S11). The PMDMs exhibited slight deviations from ideal sphericity because of mechanical stress during extrusion from microfluidics tubing postpolymerization. However, this did not affect their magnetic responsiveness or chain assembly performance (fig. S12).

This assembly capacity extended beyond individual PMDMs to preassembled PMDM chain fragments, which also combined to form a single, long chain when exposed to an oscillating magnetic field (Fig. 2H, right, and movie S4). The chain fragments first gravitated toward each other, upon which the chain fragment ends aligned and connected, culminating into one long chain that remained stable after the magnetic field was removed (fig. S13A). Scanning electron microscopy (SEM) images confirmed that the ferromagnetic portions of the PMDMs within the chain remain tightly connected (fig. S13B). The length of the PMDM chains could be tuned linearly by adjusting *N*_s_ (fig. S13, C and D).

Furthermore, a PMDM chain could be directed to form a PMDM ring by reversing the magnetic field direction when subjected to a static, horizontal magnetic field. Two ends of the PMDM chains followed the field reversal faster than the main body, approaching each other and merging to form a ring when they curved in the same direction (fig. S14). The chain-to-ring transformation was reversed by reversing the direction of the magnetic field (fig. S14).

### Multimodal locomotion of assembled PMDM chains

**Fig. 3.**
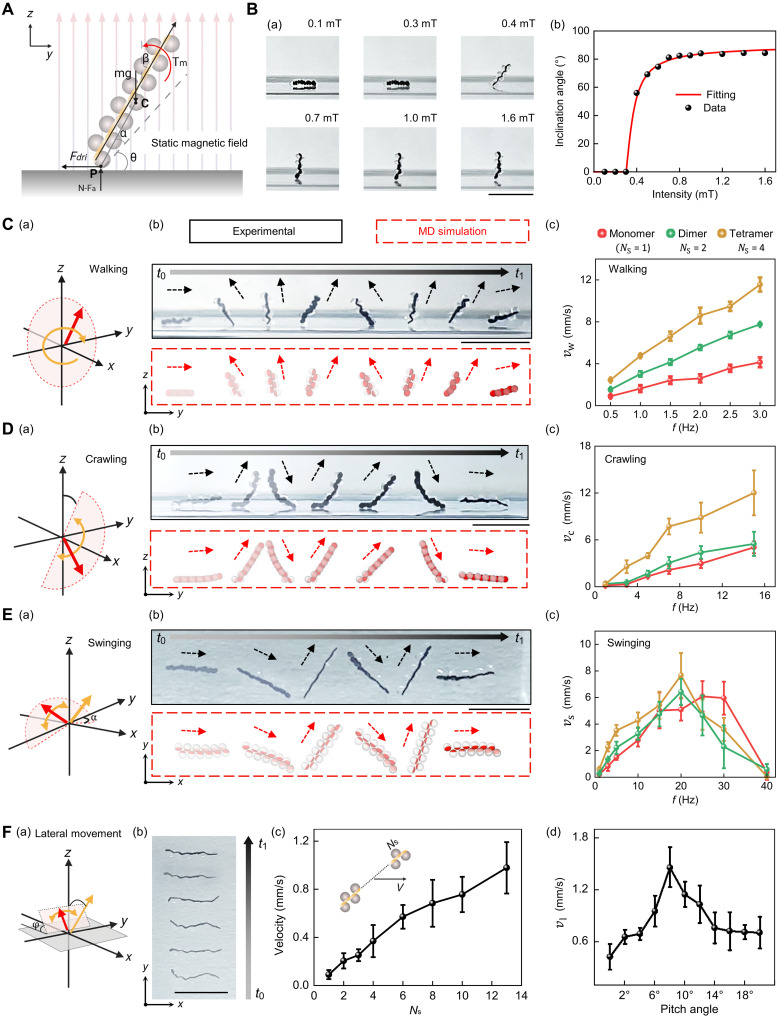
Multimodal locomotion of assembled PMDM chains. (**A**) Force diagram of an assembled PMDM chain under a uniform and static magnetic field. Point C indicates the centroid, and point P denotes the contact point. (**B**) Experimental images (a) and plot (b) depicting the relationship between the equilibrium angle θ and the applied magnetic field strength. Scale bar, 5 mm. (**C** to **E**) Multimodal locomotion of the assembled PMDM chains: walking (C), crawling (D), and swinging (E). (a) Schematic showing the magnetic field parameters. (b) Experimental time-lapse snapshots (top) and MD simulation (bottom) of each gait. Scale bars, 5 mm [*t*_0_ = 0 s in (C) to (E), *t*_1_ = 4.10 s in (C), *t*_1_ = 3.59 in (D), and *t*_1_ = 7.28 s in (E)]. Dashed arrows indicate the orientation of the robot at each time point, highlighting differences in gait patterns and body-axis alignment during motion. (c) Velocity versus actuation frequency for different chain lengths (*N*_s_ = 1, 2, and 4). Data are shown as the means ± SD (*n* = 3). (**F**) Lateral locomotion of PMDM chains. (a) Schematic showing the magnetic field parameters for lateral movement. (b) Time-lapse images of lateral translation actuated by a horizontal oscillating field (6 mT, 32 Hz). Robots do not contact the container walls during motion. Scale bar, 5 mm. (c) Locomotion velocity for chains with varying *N*_s_. Data are shown as the means ± SD (*n* = 3). (d) Mean locomotion velocity as a function of pitch angles. Data are shown as the means ± SD (*n* = 3). Created in BioRender. Cao, Y. (2025) https://BioRender.com/1tjzm3n.

Given that each individual PMDM is axially magnetized, where its magnetization **M** is oriented along the center line, the magnetization direction of the PMDM chains remains constant. As a result, when exposed to a static and vertical magnetic field **B**, the PMDM chain underwent collective rotational motion to align with the external field because of the magnetic torque induced on the embedded magnetic particles (Fig. 3A).

To determine the minimal external magnetic field required to induce rotational torque, the relationship between the rotational angle θ and the applied magnetic field intensity **B** was examined by measuring θ using image analysis. The minimum magnetic intensity $|B_{\text{min}}|$ required for the chain to rotate was determined to be 0.4 mT [Fig. 3B (a)]. At ~84° at 1.0 mT, the angle of inclination began to plateau despite a further increase in field strength [Fig. 3B (b)].

Next, we investigated the ability of PMDM chains to undergo multimodal locomotion, including walking, crawling, swinging, and lateral movement, by alternating dynamic magnetic fields. By imposing a magnetic field (4 mT) rotating in the *yz* plane (magnetic field frequency *f* = 1 Hz) [Fig. 3C (a)], the PMDM chains displayed walking behavior in the *y* direction (movie S5). By following the magnetic field, one end of the chain lifted off the surface and moved in an arc above the other end that was still in contact with the surface and fixed in place because of friction. Once the lifted end finished the arc and regained contact with the surface, now in a new location, it stayed in place, while the other end of the chain lifted off in an arc and completed the full rotation [Fig. 3C (b)]. The experimental results agree with the MD simulations [Fig. 3C (b)]. Furthermore, the velocities of the PMDM chains can be varied by modulating either the frequency *f* of the external magnetic field or the number *N*_s_ of PMDMs. The walking velocity *v*_w_ of the dimer (*N*_s_ = 2) and tetramer (*N*_s_ = 4) linearly increased with *f* [Fig. 3C (c)].

When exposed to a vertical magnetic field (6 mT) with a frequency of *f* = 3 Hz in the *yz* plane with an angular amplitude from $-\frac{2}{3}\pi$ to $\frac{1}{3}\pi$ in relation to the *y* direction [Fig. 3D (a)], the PMDM chains performed a crawling motion (movie S5). In this locomotion mode, the lifted ends did not complete the full arc but descended back to the surface instead when the rotation direction of the magnetic field was reversed [Fig. 3D (b)]. Because of the asymmetry of the precessing direction, the chain moved forward. The MD simulation also qualitatively reproduced the crawling motion [Fig. 3D (b)]. In addition, Fig. 3D (c) shows the relationship in crawling velocities *v*_c_ of the monomer (*N*_s_ = 1), dimer (*N*_s_ = 2), and tetramer (*N*_s_ = 4) under different magnetic fields *f*. Similar to what was observed for the walking behavior, increasing *f* increased *v*_c_.

While applying a horizontal magnetic field (6 mT, *f* = 2 Hz) in the *xy* plane at an angle α = −30° with an angular amplitude from −π to π in relation to the *x* direction [Fig. 3E (a)], the PMDM chain swung and moved forward along a straight line (movie S5). The MD simulation shows that the front end of the chain lifts because of α, causing a snake-like swinging motion to propel the chain forward [Fig. 3E (b)]. For the swinging motion, the PMDM chains displayed a nonlinear response in terms of swinging velocity *v*_s_ versus *f*. As *f* increased, *v*_s_ increased until it reached a threshold, referred to as the step-out frequency (*33*), and subsequently decreased for values of *f* beyond the threshold [Fig. 3E (c)].

While applying a horizontal magnetic field (6 mT, *f* = 3 Hz) in the *xy* plane at a pitch angle φ = 6° around the *x* direction with angular amplitude between $-\frac{1}{3}\pi$ and $\frac{1}{3}\pi$ in relation to the *x* direction and a rotational axis (the orange arrow) inclination angle of −30° from the *z* axis [Fig. 3F (a)], we expect the fluidic drag encountered by the two ends of the PMDM chains to be different, leading to lateral movement [Fig. 3F (b) and movie S5]. The PMDM chain moved linearly forward and backward in the *xy* plane [Fig. 3F (b)]. Notably, both the *N*_s_ of PMDMs and magnetic field frequency *f* influence the lateral locomotion velocity *v*_1_. The *v*_1_ of the PMDM chains increased with *N*_s_ [Fig. 3F (c)] and varied with *f* as in the swinging case [Fig. 3F (d)].

### Controlled locomotion on uneven surfaces and manipulation of the assembled PMDM chains

**Fig. 4.**
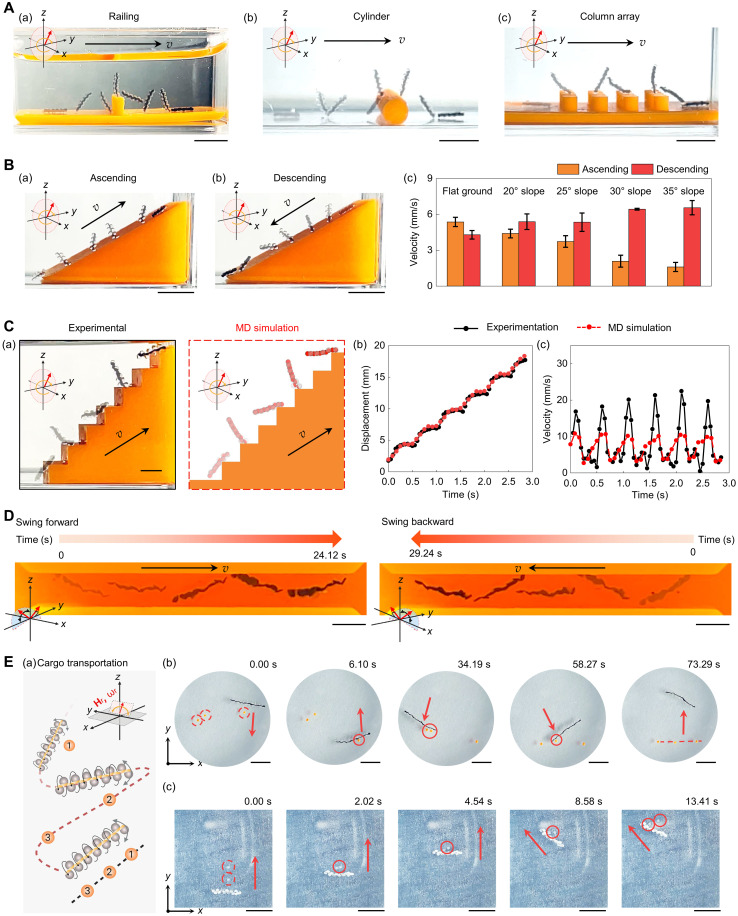
Controlled locomotion and manipulation of assembled PMDM chains across diverse terrains. (**A**) Navigation through various obstacles. (a to c) PMDM chains traversing a railing (3-mm height) (a), a cylinder (3-mm diameter) (b), and a column array (2-mm height and 2-mm spacing) (c). Scale bars, 5 mm. (**B**) Locomotion on inclined surfaces. (a and b) A chain ascends (a) and descends (b) a 30° slope. Scale bars, 5 mm. (c) Mean ascending and descending velocity of chain centroids at slope angles of 20°, 25°, 30°, 35°, and on flat ground. Data are shown as the means ± SD (*n* = 3). (**C**) A PMDM chain climbs stairs. (a) A chain ascends stairs. Scale bar, 2 mm. (b and c) Comparison of experimental and MD simulation results for the displacement (b) and the velocity profile (c) of the chain centroid. (A to C) All tests used a 4-mT vertical rotating magnetic field at 1 Hz. (**D**) Swinging motion across a 2-mm-wide channel in forward and backward directions. Scale bars, 2 mm. (**E**) Manipulation of cargo. (a) Schematic of the lateral transport of PEGDA microspheres (yellow). (b) Manipulation of randomly placed PEGDA microspheres (*D* = 600 μm, orange). The red dotted and solid line circles indicate the initial and intermediate microsphere positions, respectively. The red arrows indicate the movement direction. Field: 6 mT, 1 Hz, 6° pitch angle. Scale bars, 5 mm. (c) Transport of hMSC-laden Matrigel microspheres. Red markings indicate the position and motion. Field: 7.9 mT, 1 Hz, 6° pitch angle. Scale bars, 5 mm. Created in BioRender. Cao, Y. (2025) https://BioRender.com/ondsoh4.

Given the ability to control the PMDM locomotion mode, we next sought to determine whether this would enable the microrobots to traverse challenging or complex environments. The magnetic coupling strength between the individual PMDMs within a chain allowed them to overcome different obstacles across various terrains without breaking apart, including a railing (3-mm height), a cylinder (3-mm diameter), and a column array (2-mm height and 2-mm distance) (Fig. 4A and movie S6). To further evaluate their ability in navigating obstacles, we tested the walking motion of the PMDM chains on inclined surfaces with different slopes and stairs. PMDM chains demonstrated proficiency in both climbing and descending inclines, successfully navigating slopes with angles of 20°, 25°, 30°, and 35° [Fig. 4B (a and b) and movie S7]. As expected, the mean ascending velocity of the PMDM chains decreased as the slope of the incline increased [Fig. 4B (c)].

PMDM chains can also ascend and descend stairs (2-mm gap and 2-mm increasing height), as verified experimentally and by using MD simulation [Fig. 4C (a), fig. S15A and movie S6]. The displacement and instantaneous velocity curves from the MD simulation matched the experimental results [Fig. 4C (b and c) and fig. S15, B and C], demonstrating the accuracy and predictive power of MD simulation for optimizing microrobot design.

We can influence the ascending velocity of the PMDM chains by modifying the rotating frequency of the magnetic field. PMDM dimers with a body length of 1 mm, for example, achieved the fastest climbing speed of 9.85 mm/s when we increased the frequency of the rotating magnetic fields to 10 Hz (fig. S16, A and B, and movie S8). By comparing the relative climbing speed with respect to body length between PMDMs and other reported microrobots, PMDMs achieved a climbing speed of 9.85 body lengths per second, marking the fastest climbing speed at the submillimeter scale reported to date (fig. S16C and table S1).

Whereas PMDM chains overcome tall obstacles by walking, they traverse confined spaces by swinging. PMDM chains with a 1-mm width and a 2-mm body length can swing forward and backward within a 2-mm-wide channel (Fig. 4D and movie S6).

To showcase superior motion control and manipulation capabilities on the basis of lateral movement, we used PMDM chains to manipulate both rigid PEGDA microspheres and soft Matrigel microgels. We repositioned randomly distributed PEGDA microspheres into a linear sequence [Fig. 4E (a and b) and movie S9]. In addition, the PMDM chains successfully demonstrated the transport of human mesenchymal stem cell (hMSC)–laden Matrigel microgels without compromising cell viability, as shown in Fig. 4E (c), fig. S17, and movie S9. These results highlight the potential of PMDM chains for transporting and delivering cell-laden microgels to specific sites for disease treatment in vivo.

### Adaptive locomotion of PMDM chains with reconfigurable transformation

Introducing a large number of microrobots into the system enables the formation of swarm microrobots, which are capable of dynamic shape transformation, allowing them to navigate through challenging environments (*1*, *34*). By applying forces that exceed the dipole forces holding PMDMs together, the chains disassemble into individual PMDMs or shorter-chain fragments. To achieve this in our system, we increased the centrifugal force experienced by the PMDM chains by increasing the frequency of the rotating magnetic field, leading to their spontaneous disassembly into dispersed PMDMs (fig. S18). By subsequently decreasing the frequency, the PMDMs reassembled back into longer chains (fig. S18). The frequency-induced transformation between assembled and disassembled swarms is rapid and reversible, enabling a high degree of control over their effective motions.

**Fig. 5.**
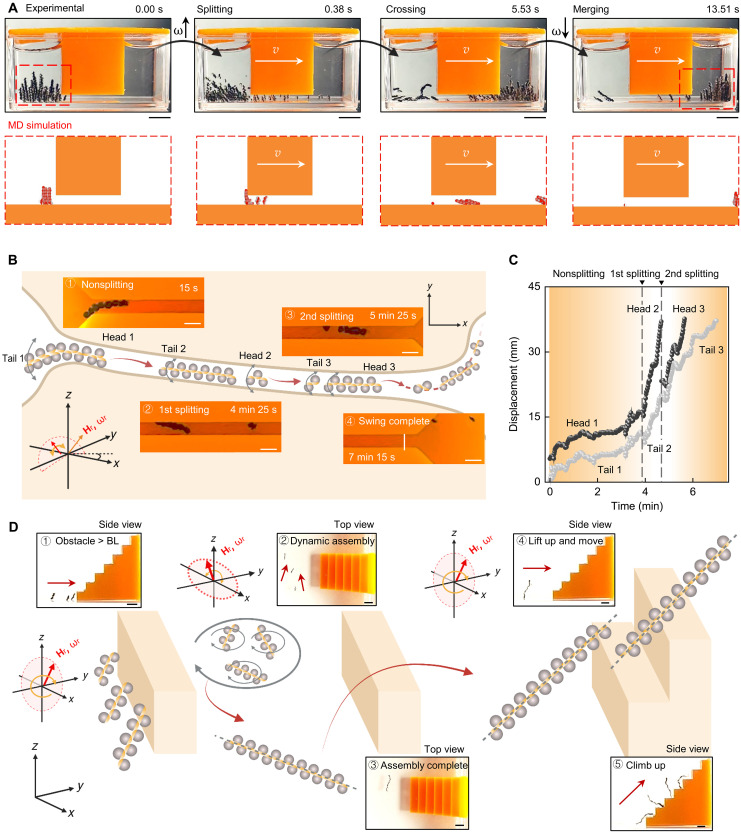
Reconfigurable transformation and adaptive locomotion of assembled PMDM chains and swarms. (**A**) Experimental and MD simulation snapshots showing assembled PMDM swarms successfully crossing a 2-mm gap. Scale bars, 5 mm. (**B**) Schematic illustration and sequential images of a PMDM chain navigating through a 1.5-mm-wide channel. The chains disassembled into shorter PMDM chain segments that moved through the confined space. Scale bars, 2 mm. (**C**) Positional displacements of the head and tail segments of the assembled PMDM chain over time while traversing a narrow channel in three distinct phases: nonsplitting, first splitting, and second splitting. (**D**) Schematic illustration and sequential images of PMDM chain fragments climbing over stairs with heights exceeding the chain length. The PMDM chain fragments amalgamate, facilitating successful traversal. Scale bars, 2 mm. Created in BioRender. Cao, Y. (2025) https://BioRender.com/8huwn9o.

We illustrated this both in vitro and in silico by placing PMDM chain swarms into an environment consisting of two chambers connected by a narrow channel, as shown in Fig. 5A and movie S10. Initially, the PMDMs were placed inside the left chamber and assembled into multiple chains under a low-frequency rotating magnetic field (*f* = 1 Hz) (Fig. 5A). Afterward, the swarm walked toward the channel, where the smaller PMDM chains that fit the channel height moved to the right chamber. Chains longer than the channel height, however, hit against the chamber wall and stayed in the left chamber. To induce the disassembly, the magnetic field frequency was switched to 20 Hz, causing the PMDM chains to quickly (within 0.38 s) disassemble into fragments up to five times smaller than their original body length. The smaller chain fragments were able to pass through the narrow channel (Fig. 5A). After all the PMDMs reached the right chamber, the field frequency was reverted to 1 Hz and the chain fragments reassembled into longer PMDM chains.

We also observed that the PMDM chain swarms could move collectively along a defined path, such as a circular trajectory (fig. S19A and movie S11). The large number of PMDMs in the swarm generated substantial forces and kinetic energy during their motion, which could be harnessed for transporting heavy cargo. As shown in fig. S19B and movie S11, the PMDM chain swarms successfully transported a ring weighing 450 mg, which is ~3000 times the mass of a single PMDM (mass, 0.15 mg).

Given the versatility of the PMDMs, we sought to leverage their ability to spontaneously disassemble on demand to pass through a narrow channel. We designed a channel (where the width ratio between the PMDM chain and the channel was 70%) that would purposefully limit the efficiency of a swinging mode of travel (Fig. 5B). Initially, the PMDM chain struggled to swing through the confined channel because the swinging motion required ample space for the tail to generate propulsion (movie S10). Because of the limited space, the velocity of the PMDM chain remained relatively low, hindering the movement (Fig. 5C). To overcome this, we split the chain twice by increasing the field frequency, allowing the shorter-chain segments to successfully traverse through the channel.

A longstanding challenge for microrobot locomotion has been surpassing vertical obstacles much taller than their height. To overcome such barriers, we used the PMDMs’ adaptive locomotion whereby multiple shorter PMDM chain fragments assembled into a long PMDM chain under a horizontal oscillating magnetic field (Fig. 5D and movie S10). Once assembled, this elongated PMDM chain had sufficient length to climb tall obstacles by switching to a walking movement mode (Fig. 5D). This adaptive locomotion enhanced the functionality of the robotic system, allowing the microrobots, in challenging environments, to autonomously decide on appropriate actions (*34*, *35*).

### Delivery of PMDMs in biological environments

**Fig. 6.**
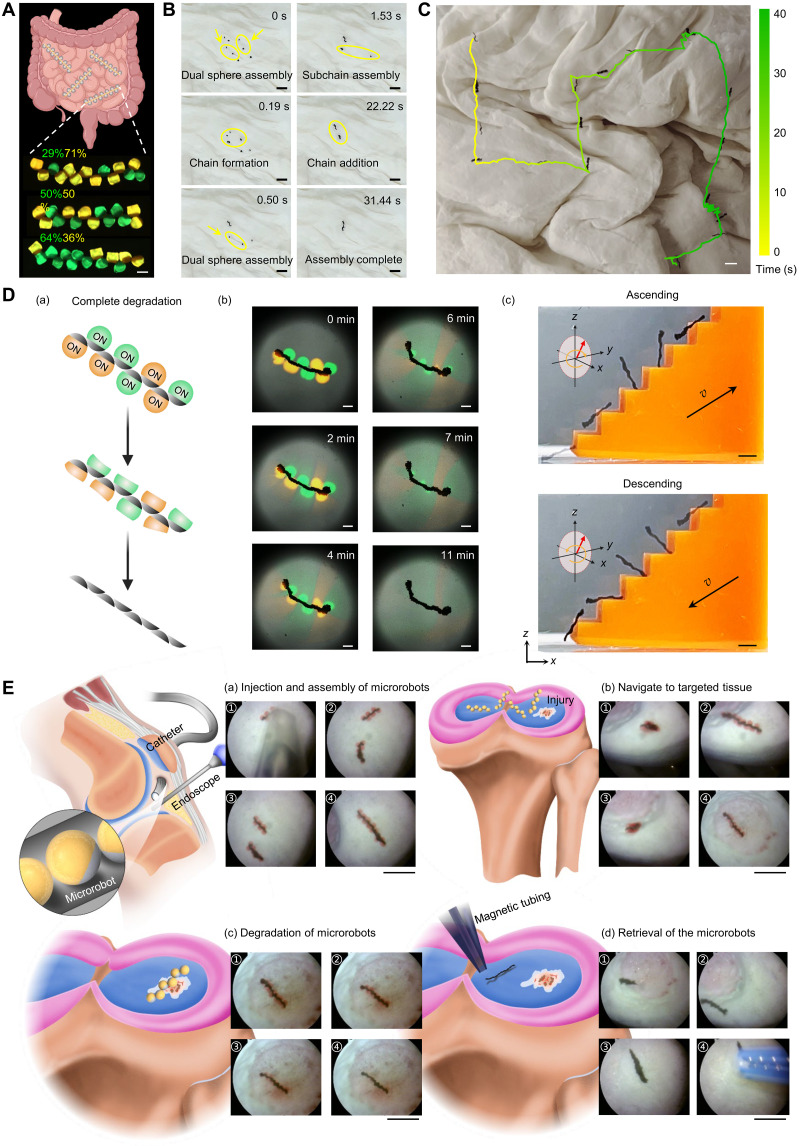
Delivery of PMDM chains in biological environments. (**A**) Schematic showing PMDMs for programmable drug delivery. Green and yellow fluorescent PMDMs represent carboxylate-modified FluoSpheres microspheres (*D* = 0.2 μm, Invitrogen) labeled with different fluorophores to simulate distinct cargo types. Each type was loaded at a concentration of 20 μl/ml into an 80% (v/v) PEGDA hydrogel matrix containing 25% (w/v) NdFeB microparticles. Scale bar, 500 μm. (**B**) Time-lapse snapshots showing the stepwise assembly of PMDMs (dimers, fragments, and full chains) on an uneven porcine intestine surface. Scale bars, 3 mm. (**C**) Locomotion of a PMDM chain across ex vivo intestinal tissue under magnetic actuation. Scale bar, 5 mm. (**D**) Controlled dual cargo release. (a and b) Schematic (a) and chronological series of images (b) showing the release of the dual FluoSpheres carboxylates from the assembled PMDM chain. Scale bar, 500 μm. Green: *D* = 0.2 μm, 20 μl/ml, dissolved within a composite matrix that includes 1% (w/v) alginate-7.5% (w/v) gelatin with 25% (w/v) NdFeB microparticles. Yellow: *D* = 0.2 μm, 20 μl/ml, dissolved within a composite matrix that includes 1% (w/v) alginate-5% (w/v) gelatin with 25% (w/v) NdFeB microparticles. (c) Degraded chains retain structural integrity and climb/descend stairs. Scale bars, 2 mm. (**E**) Delivery of preassembled PMDM chains into a printed human cartilage model. The whole procedure consists of injections (a), assembly (a), locomotion (b), drug release (c), and retrieval (d) of PMDMs. Scale bars, 5 mm. Figure produced using SolidWorks and Inkscape. Schematics in (A) and (D) created in BioRender. Cao, Y. (2025) https://BioRender.com/pvwbk8n.

Precisely delivering diverse combinations of cargoes in varying proportions is advantageous for biomedical applications. The ability to design PMDMs with different hydrogel compositions suitable for the delivery of different types of cargoes creates the opportunity to design bespoke solutions. For instance, we can control the cargo dosage by adjusting the number of PMDMs loaded with cargo. To illustrate the concept, we fabricated PMDMs loaded with green FluoSpheres carboxylates (diameter, 0.2 μm; 5.0 μl/ml) or yellow FluoSpheres carboxylates (diameter, 0.2 μm; 1.0 μl/ml) in PEGDA hydrogel to represent different cargo types. These PMDMs were assembled into chains with multiple configurations (Fig. 6A and fig. S20A). Furthermore, we demonstrated that PMDMs can simultaneously carry multiple cargoes by combining different hydrogel matrices. Here, we demonstrated the concept using soft alginate/gelatin hydrogels for cell delivery and rigid PEGDA for yellow fluorosphere carboxylate delivery (fig. S20B).

Biocompatibility is an important prerequisite for translating to clinical applications. We used human umbilical vein endothelial cells (HUVECs) to evaluate the PMDM cytocompatibility and quantified the cell viability over 7 days of culture. Compared with the pure NdFeB microparticles, the PMDMs did not adversely affect cell viability (fig. S21).

We also evaluated the delivery of PMDMs in intricate biological environments using ex vivo porcine intestine and a 3D-printed human cartilage model. After delivering PMDMs (composed of gelatin/alginate hydrogel and loaded with green and yellow FluoSpheres carboxylates) inside a porcine intestine surface containing rugae and mucosa, the microrobots successfully assembled into chains by chain fragment addition and chain assembly, the assembly processes discussed in previous sections (Fig. 6B and movie S12). This indicates that the uneven tissue surface does not affect the chain assembly efficiency. Once assembled, the PMDMs exhibited effective self-adaptive locomotion on the compliant, viscous, and unstructured intestine surface (Fig. 6C and movie S12), following a predetermined path. This observation highlights the potential of using PMDMs to navigate through complex biological environments for the precise delivery of therapeutic agents.

After the PMDMs reached the target site, we achieved a controlled dual release of the two cargo types by submerging the PMDMs in a phosphate-buffered saline (PBS) solution containing collagenase type I (2 mg/ml) at 37°C [Fig. 6D (a) and movie S13]. First, PMDM chains containing green and yellow fluorescent microspheres were fabricated using two different hydrogel formulations: 1% (w/v) alginate with either 5 or 7.5% (w/v) gelatin. We then evaluated the release dynamics of model drugs using a fluorescence area–based analysis. As shown in Fig. 6D (b) and fig. S22, both formulations exhibited a burst release profile. The 5% (w/v) gelatin formulation degraded more rapidly, reaching near-complete release within ~6 min, while the 7.5% (w/v) gelatin matrix showed a slower degradation rate and delayed release (fig. S22). Even after full degradation of the hydrogel phase and the complete release of the FluoSpheres carboxylates, the chain assembly retained its integrity and remained intact. Moreover, the degraded PMDM chain maintained its locomotion capability like those of the nondegraded PMDM chains and could still assemble and overcome obstacles [Fig. 6D (c), fig. S23, and movie S13]. This suggests that after completing the cargo delivery, the ferromagnetic microparticles can be fully retrieved, offering a safer alternative to other magnetic microrobots, where magnetic particles typically detach from the hydrogel (*20*) and are difficult to retrieve. The retention of the ferromagnetic microparticles is likely due to their larger size (5 μm), which exceeds the pore size of the hydrogel network, and to the strong intrinsic magnetic multipolar forces between the ferromagnetic microparticles. We further verified this biosafety advantage through degradation-recovery experiments on porcine intestine and glass slides. SEM and EDS mapping confirmed that NdFeB microparticles remained clustered after degradation and were fully retrieved, with no detectable Fe or Nd signal remaining on the substrate within the detection limits of the technique (fig. S24).

To further demonstrate programmable release, we constructed hybrid PMDM chains composed of rigid PEGDA segments (loaded with yellow FluoSpheres carboxylates) and degradable alginate/gelatin segments (loaded with green FluoSpheres carboxylates) (fig. S25A). Upon exposure to collagenase, only the green-labeled cargo from the degradable domains exhibited burst release, while the yellow-labeled PEGDA segments showed minimal release over the same timescale (fig. S25A). Quantitative fluorescence area analysis confirmed this material-dependent and spatially controlled release profile (fig. S25B). These results indicate that the much slower degradation of PEGDA allows for extended cargo retention and delayed release.

Also, the partially degraded hybrid PMDM chains maintained their structure and traversed complex obstacles, including ascending and descending stairs (fig. S25C). The assembly of two hydrogel materials through ferromagnetic interactions provides a method for creating hybrid PMDMs capable of multiple, controlled drug releases. The locomotion capacity of hybrid PMDM chains will be beneficial for targeted sequential release of different drugs at different locations.

To demonstrate the potential clinical application of the platform, we developed an endoscope-assisted technique to deliver PMDMs to a 3D-printed human cartilage with an injury site (fig. S26 and movie S14). First, we loaded the PMDMs or preassembled PMDM chain fragments into a catheter [Fig. 6E (a)]. We used catheters of different diameters to deliver PMDM chains with varying numbers of PMDMs. After injection, we applied an oscillating magnetic field to assemble the PMDMs into a cohesive chain [Fig. 6E (a)]. The assembled chain then adapted its locomotion mode to navigate to the targeted injury site [Fig. 6E (b)]. Upon arrival, the designed degradation properties of the hydrogel matrix led to drug release [Fig. 6E (c)]. After drug delivery, we navigated the degraded ferromagnetic chain to the initial injection site and retrieved the microrobots using a catheter equipped with small magnets [Fig. 6E (d)].

## DISCUSSION

We have developed highly controllable and multifunctional PMDMs using droplet microfluidics. We demonstrated experimentally and computationally that PMDMs exhibit assembly, disassembly, and reassembly; can adopt four different modes of adaptive and effective locomotion; and are capable of navigating through various structured and unstructured terrains. Furthermore, the reconfigurable nature of PMDM chains enabled them to adapt to challenging environments, such as confined narrow spaces and high obstacles. When grouped in larger numbers, the PMDMs formed swarm configurations that displayed collective behaviors, such as shape-shifting to traverse narrow confinements and the ability to transport sizable cargo. The combination of experimental and computational data provides a toolbox for efficiently predicting their dynamic behaviors and customizing the design of magnetic microrobots in the future.

Building on these capabilities, the inherent modularity and the hydrogel composition of the PMDMs allow for the controlled and precise delivery of various cargo combinations. In our study, we successfully released cargo by using enzymes to break down the material. By incorporating other responsive materials sensitive to temperature or pH levels, we can achieve on-demand release. The magnetic phases allow the formation of hybrid, multimaterial chains capable of delivering different cargoes in different modes of locomotion. In contrast, traditional microrobots are typically limited to a single type of cargo (*25*), restricting their application in in vivo environments.

These capabilities position PMDMs as promising candidates for clinical use in disease contexts that require spatially and temporally resolved delivery of drugs. For example, in inflammatory bowel disease, it often requires localized administration of multiple drugs (e.g., corticosteroids, immunomodulators, and regenerative factors) at different sites of inflammation along the intestine (*36*). Similarly, solid tumors, such as colorectal and pancreatic cancers, may benefit from sequential and localized delivery of chemotherapeutics, immunotherapeutics, or matrix-degrading enzymes to optimize tissue penetration and therapeutic efficacy in heterogeneous tumor microenvironments (*37*).

To ensure intestinal targeting, we demonstrated endoscope-assisted injection delivery in ex vivo porcine intestines. This clinically established method bypasses the acidic gastric environment. For future potential in the oral delivery of drugs, we could encapsulate PMDMs in pH-sensitive enteric coatings that remain stable in the stomach but dissolve in the intestines (*38*).

PMDMs are actuated by external magnetic fields and can be integrated into advanced electromagnetic actuation (EMA) platforms. Notably, the OctoMag system developed by Kummer *et al.* (*39*) provides six degrees of freedom in microrobot positioning using eight stationary electromagnets. This system has been successfully applied in confined and delicate anatomical sites, such as the eye and vascular system (*40*). Beyond hardware, recent developments in reinforcement learning and closed-loop control algorithms offer potential to improve microrobot navigation accuracy and adaptability (*41*, *42*). Reinforcement learning–based controllers can dynamically modulate magnetic fields on the basis of real-time sensory input to optimize path planning, obstacle avoidance, and target engagement in complex biological environments. Integrating PMDMs with such intelligent control schemes may enable future autonomous or semiautonomous microrobot operation. For real-time tracking, magnetic particle imaging provides high-resolution, deep-penetration, and background-free imaging of magnetic microrobots (*43*, *44*). Combining magnetic particle imaging with closed-loop magnetic actuation forms a powerful framework for intelligent and image-guided therapy. Additional modalities such as ultrasound, fluorescence, x-ray, or endoscopic cameras may be incorporated for assisting multimodal localization during clinical procedures.

Traditional microrobots often struggle to retrieve magnetic parts from living environments after cargo release (*45*), as magnetic particles can disperse and linger, potentially triggering unwanted immune responses. In contrast, the PMDM chains maintained structural integrity and movement capabilities even after the hydrogel phase fully degraded. This ensures that they can be completely removed from the host environment, enhancing the overall safety of the system. We demonstrated their ability to deliver and move within a 3D-printed human cartilage model and a pig intestine, confirming that they can function effectively in complex biological settings. In essence, the PMDM platform stands out as a promising avenue for clinical interventions, offering enhanced functionalities, superior navigational abilities, and minimal invasiveness, all of which will minimize patient discomfort and maximize therapeutic outcomes.

## MATERIALS AND METHODS

### Fabrication of PMDMs

PMDMs were made via cascade tubing microfluidics, a previously reported system for mass production of microspheres (*46*). The microfluidic system encompassed two distinct phases: the ferromagnetic composite hydrogel phase and the hydrofluoroether (HFE) 7100 oil phase. To prepare rigid PEGDA PMDMs, 2% (w/v) lithium phenyl(2,4,6-trimethylbenzoyl)phosphinate was dissolved in the 80% (v/v) PEGDA hydrogel phase (molecular weight, 700 Da; Sigma-Aldrich) and sonicated for 10 min at room temperature. NdFeB microparticles (25%, w/v) with an average size of 5 μm were added into the PEGDA hydrogel and homogeneously dispersed using a vortex generator (Thermo Fisher Scientific) at 3000 rpm for 2 min. Next, the ferromagnetic composite hydrogel was loaded in a 1-ml syringe and transported through a polytetrafluoroethylene tubing [500-μm inside diameter (ID) and 1-mm outer diameter (OD)] to a handmade three-way polydimethylsiloxane connector by a syringe pump (Harvard Apparatus). The HFE 7100 oil phase was loaded in a 10-ml syringe and pumped to the connector using another syringe pump (Harvard Apparatus). Within the three-way connector, PEGDA droplets were formed and collected in a separate piece of tubing (500-μm ID and 1-mm OD). The flow rates of the ferromagnetic composite hydrogel and oil phases were set at 30 and 150 μl/min, respectively. The collection tubing containing PEGDA droplets was left in air for 30 min to allow for complete sedimentation of the NdFeB microparticles. Subsequently, the droplets were cross-linked by exposure to an ultraviolet lamp with a wavelength of 365 nm for 20 min. Soft alginate/gelatin PMDMs were fabricated following the procedures described previously. Briefly, 1% (w/v) alginate combined with either 5 or 7.5% (w/v) gelatin and 25% (w/v) NdFeB microparticles was prepared as the ferromagnetic hydrogel precursor. The alginate/gelatin droplet generation process was carried out at 37°C to prevent the gelation of the alginate/gelatin phase. After that, the collection tubing containing alginate/gelatin droplets was left in an incubator at 37°C for 10 min to allow for complete sedimentation of the NdFeB microparticles. Then, the collection tubing was left in a fridge at 4°C overnight to thermally cross-link, followed by full cross-linking by soaking in a saturated transglutaminase solution and 0.2 M CaCl_2_ solution. The fabricated PMDMs were then washed using PBS and stored in deionized water at 4°C.

### Computational particle model

The PMDMs are described by their geometrical center position $r_{c}$ , their normalized orientation vector u pointing in the direction of their symmetry axis, and the center of mass of their magnetic part $r_{m}=r_{c}-s\cdot u$ , with $s=\frac{6{\left(1-h\right)}^{2}R}{2\left(3-2h\right)}$ being the distance between $r_{c}$ and $r_{m}$ (fig. S6). We used the two different positions to replicate the anisotropic mass distribution and the accurate ferromagnetic response to an external magnetic field **B**. For the latter, we assigned a dipole moment μ⊥u to the position $r_{m}$ . We modeled the glass interfaces and all obstacle objects as hard walls with normal direction **n**.

The excluded volume interactions between the particles are modeled by the purely repulsive Weeks-Chandler-Andersen (WCA) potential

$$\begin{array}{c} \begin{array}{c} U_{\text{WCA}}\left(r_{c,\mathrm{ij}}\right)=\left\{4\varepsilon \left[{\left(\frac{\sigma }{r_{c,\mathrm{ij}}}\right)}^{12}-{\left(\frac{\sigma }{r_{c,\mathrm{ij}}}\right)}^{6}\right]-\varepsilon ,r_{c,\mathrm{ij}}<\sqrt[{6}]{2}\sigma 0,\;\text{otherwise}\;\right. \end{array} \end{array}$$ (1)

with particle center distance $r_{c,\mathrm{ij}}=\|r_{c,i}-r_{c,j}\|$ and the energy unit ε=1 , which represents 4.0694 × 10^−10^ J. The dipole-dipole forces that stem from the magnetized lower part of the particles are modeled by the interaction potential

$$U_{\text{dipole}}\left(r_{m,\mathrm{ij}}\right)=\varepsilon \left[\frac{\mu_{i}\cdot \mu_{j}}{{r_{m,\mathrm{ij}}}^{3}}-3\frac{\left(\mu_{i}\cdot r_{m,\mathrm{ij}}\right)\left(\mu_{j}\cdot r_{m,\mathrm{ij}}\right)}{{r_{m,\mathrm{ij}}}^{5}}\right]$$ (2)

Here, the magnetic dipole moments $\mu_{i}⊥u_{i}$ and $\mu_{j}⊥u_{j}$ are located at $r_{m,i}$ and $r_{m,j}$ , respectively, and the magnetic center of mass distance $r_{m,\mathrm{ij}}=\|r_{m,ij}\|=\|r_{m,i}-r_{m,j}\|$ . The magnetic dipoles also interact with the external magnetic field **B** via the potential $U_{B}\left(r_{m,i}\right)=B\cdot \mu_{i}$.

To include the effects of gravity and buoyancy, we apply a constant force $G_{c,i}=-\left(\rho_{\text{PEGDA}}-\rho_{H_{2}O}\right)V_{\text{sphere}}g\cdot z$ to $r_{c,i}$ and $G_{m,i}=-$ $\left(\rho_{\text{NdFeB}}-\rho_{\text{PEGDA}}\right)V_{\text{cap}}g\cdot z$ to $r_{m,i}$ pointing in the −***z*** direction with the densities $\rho_{H_{2}O}=1.0g/\text{ml}$ , $\rho_{\text{PEGDA}}=1.12g/\text{ml}$ , and $\rho_{\text{NdFeB}}=$ 7.6g/ml, the volumes of the spherical particle $V_{\text{sphere}}=\frac{1}{6}\pi \sigma^{3}$ and the magnetic sphere cap $V_{\text{cap}}=\frac{1}{6}\pi h^{2}\left(3-2h\right)\sigma^{3}$ , and the gravity constant *g*. We model the interface as a planar wall with the normal direction ***z***. In addition to WCA-type interactions with the particle, the wall also applies static and dynamic friction to the system components, which creates a counteracting force $\lambda F_{\text{friction}}$ perpendicular to and torque $\lambda T_{\text{friction}}$ (anti)parallel to *z*. Here, we use $\lambda_{\text{static}}=0.5$ and $\lambda_{\text{dynamic}}=0.4$ for the static and dynamic friction coefficients when the particles are in a chain arrangement. For individual particles, we neglect friction completely as, in that case, the particles roll instead of slide with a smaller $\lambda_{\text{dynamic}}\gg \lambda_{\text{roll}}$.

The equations of motion of each PMDM are

$$\gamma {\dot{r}}_{c,i}=\sum_{j}\left(F_{\text{WCA},\mathrm{ij}}+F_{\text{dipole},\mathrm{ij}}\right)+G_{c,i}+G_{m,i}^{F}+F_{\text{Wall},i}-F_{\text{friction},i}$$ (3)

$$\gamma_{r}{\dot{\omega }}_{i}=\sum_{j}T_{\text{dipole},\mathrm{ij}}+G_{m,i}^{T}+T_{B,i}-T_{\text{friction},i}$$ (4)

The equations model nonthermal Brownian dynamics where γ = 0.3 and $\gamma_{r}=\frac{\sigma^{3}\gamma }{3}$ to avoid numerical instabilities.To support the simulation assumptions with physical estimates, we quantified key force magnitudes: $F_{\text{mag}}=\frac{3\mu_{0}{\mu_{i}}^{2}}{\pi d^{4}}$ . With μ_0_ = 1.25663706 × 10^−6^ m·kg s^−2^ A^−2^, the measured magnetic dipole strength μ*_i_*= 1.08 × 10^−6^ A·m^2^, and *d* = 4.8 × 10^−4^ m, we estimate $F_{\text{mag}}\approx 26\mathrm{\mu N}$ . For comparison, the combined gravitational and buoyant forces acting on a single PMDM [particle mass *m*_PMDM_ ~ 0.314 mg and displaced water mass *m*_water_ = 4/3π(300 × 10^−6^ m)^3^ × 1000 kg m^−3^ = 0.113 mg] are estimated to be *F*_g_ = 9.81 kg ms^−2^ × (*m*_PMDM_ − *m*_water_) = 1.97 μN, making them approximately an order of magnitude smaller than the magnetic forces.

We also estimated the hydrodynamic drag force using a drag coefficient of γ = 5.65 × 10^−6^ kg/s (based on Stokes flow assumptions and experimental parameters) and the highest experimentally measured PMDM velocity of *v* ~ 20 mm/s (Fig. 3). This gives a drag force on the order of *F*_d_ = γ × *v* ~ 0.1 μN, which is much smaller than both the magnetic and gravitational/buoyancy forces.

For each simulation, we ran independent replicates on one central processing unit for a time period *t* = 600τ with time step Δ*t* = 0.001τ and unit time $\tau =\frac{1}{60}s$ . We added surface friction forces to the open-source MD software HOOMD-blue (version 4.4.0) to perform our simulations (*47*) and used the Freud data analysis package for analysis (*48*) and the Signac software package for data management (*49*).

### Magnetization of PMDMs

PMDMs were magnetized using an impulse magnetizer (IM-10-30, ASC Scientific) with an impulse magnetic field of about 2.5 T. The M-H hysteresis loop was measured using a Princeton dual-head vibrating sample magnetometer at room temperature, with a single PMDM tested per session. To ensure accurate and artifact-free measurements, individual PMDMs were immobilized on the vibrating sample magnetometer sample holder using a thin layer of adhesive to prevent any rotational or translational motion during the test. The M-H hysteresis loop was obtained by sweeping the magnetic field between −1.2 and +1.2 T. The remanent magnetization was obtained when the applied magnetic field was zero.

The magnetic field distributions were simulated using commercial FE analysis software (COMSOL Multiphysics 5.4). The magnetization parameter was defined as *M* = 9.8 kA/m, which was determined through experiments on PEGDA samples containing 25% (w/v) NdFeB microparticles.

### SEM and EDS mapping of the PMDMs

The PMDMs were freeze dried and sputter coated with 15-nm chromium and then imaged by SEM (JEOL 6010LA). EDS (EDS mapping) analysis was also performed to investigate the distribution and content of elements (O, Fe, and Nd) on the PMDMs.

### Magnetic actuation of PMDM chains

The movement of PMDM chains was controlled using an EMA system composed of eight electromagnetic actuator coils (MFG-100; Magnebotix). To control the crawling, walking, and swinging of PMDM chains, a vertical oscillating magnetic field, vertical rotating magnetic field, and conical magnetic field, respectively, were used.

### Cell culture

Mesenchymal stem cells (Lonza, PT-2501) and HUVECs (PromoCell, C-12203) were cultured using Dulbecco’s modified Eagle’s medium (Gibco) and endothelial cell growth medium-2 (EGM-2) bullet kit (Lonza, CC-3162), respectively, at 37°C and 5% CO_2_. Confluency was maintained at around 50 to 90%. The medium was refreshed every 48 hours. Cells before passage 8 were used.

### Fluorescent polystyrene microsphere release and quantification

To investigate the release of fluorescent polystyrene microspheres, 20 μl/ml of either yellow or green fluorescent polystyrene microspheres (0.2 μm, Invitrogen) was dispersed in the hydrogel precursor solution comprising 1% (w/v) alginate combined with either 5 or 7.5% (w/v) gelatin. NdFeB microparticles (25%, w/v) were then homogeneously dispersed in the hydrogel. To release the encapsulated fluorescent microspheres, the assembled PMDM chains were placed in collagenase solution (2 mg/ml) at 37°C. The release process was recorded by a Zeiss Axio Observer (Carl Zeiss, Oberkochen, Germany) using the time series function. The resulting series of pictures was processed using FIJI (version 2.9.0/1.54f). Images from individual fluorescence channels and merged views were exported after adjusting brightness and contrast uniformly across samples.

To evaluate the release kinetics, we used an image-based analysis method by tracking the fluorescence area of each PMDM over time. Because the fluorescent microspheres were tightly encapsulated within the hydrogel matrix, their release was assumed to directly correlate with the degradation of the microrobot body. Therefore, the decrease in the fluorescence area of the microrobot body was used as an indirect indicator of degradation, and the cumulative release of the cargo was calculated as the inverse of the normalized fluorescence area. Specifically, the fluorescent region corresponding to the PMDM was segmented using thresholding in FIJI/ImageJ. At each time point, the fluorescence area was measured. The cumulative release percentage at each time point was defined as

$$\text{Release}\left(\%\right)=\frac{A_{0}-A_{t}}{A_{0}}\times 100$$ (5)

where *A_t_* is the microrobot’s fluorescence area at time *t*, and *A*_0_ is the initial area. Results are reported as the means ± SD.

### PMDM locomotion on ex vivo pig intestinal tissue

Specifically, fresh porcine intestines were obtained from a local food supplier in the United Kingdom. The tissue was gently emptied and rinsed with water to preserve the native mucus layer. Segments of the intestine were cut and placed in a water tank to serve as the testing substrate. Eight PMDMs were individually positioned on the intestinal surface and subsequently assembled into a chain using an oscillating magnetic field (8 mT, 60 Hz, *xy* plane). After assembly, a rotating magnetic field (10 mT, 1 Hz, *yz* plane) was applied to actuate locomotion. Both magnetic fields were generated using a commercial EMA system (MFG-100, Magnebotix). The motion of PMDMs was recorded in real time using a top-mounted digital camera. Video recordings were analyzed by open-source software Tracker version 6.1.3 (https://physlets.org/tracker/), and the trajectories were reconstructed by tracking the center of the PMDM chain to extract frame-by-frame positional data. All experiments were conducted within 24 hours of tissue collection, with the samples stored at 4°C until use.

### Cell encapsulation

For cell encapsulation, 5.0 × 10^6^ hMSCs/ml were blended into a hydrogel solution of 1% (w/v) alginate and 5% (w/v) gelatin. Before encapsulation, hMSCs were stained using CellTracker Green CMFDA (1:250 μg/ml diluted in cell culture media) for 30 min. The status of the cells encapsulated in the microrobots was observed using the Zeiss Axio Observer (Carl Zeiss, Oberkochen, Germany).

### Fabrication of hMSC microgels

The hMSC microgels were fabricated on the basis of a previously reported work (*33*). In summary, the hMSCs were mixed with 100% Matrigel solution (356243, Corning) at a cell density of 1.0 × 10^7^ cells/ml and kept at 4°C before loading into tubing. The Matrigel precursor flowed at 30 μl/min, while the HFE 7100 oil phase was at 150 μl/min. The microgels were collected in a polytetrafluoroethylene tubing (500-μm ID or 200-μm ID and 1-mm OD) and then solidified at 37°C. Afterward, they were dispersed in a petri dish with cell culture media.

### LIVE/DEAD staining

The hydrogel samples were rinsed three times with PBS buffer. Subsequently, the samples were immersed in 1 ml of Dulbecco’s PBS solution containing Calcein AM Viability Dye (0.5 μl/ml) for live cell staining (Thermo Fisher Scientific, C0875) and ethidium homodimer-1 (2 μl/ml; Thermo Fisher Scientific, E1169) for dead cell staining for 30 min, followed by gentle PBS buffer washing. All microscopy images were captured under a Zeiss Axio Observer (Carl Zeiss, Oberkochen, Germany).

### Cytocompatibility of PMDMs

HUVECs were seeded at 20,000 per well in 96-well plates. Five PEGDA PMDMs were placed in each well. For the control group, 0.55 μg of pure NdFeB microparticles was added to each well. The effect of PMDMs and NdFeB powder on cell viability was evaluated for 7 days and tested using an alamarBlue assay (Thermo Fisher Scientific).

### Motion analysis

The videos showcasing various microrobot motion patterns were acquired using a digital camera. These videos were subsequently analyzed by open-source software Tracker version 6.1.3 (https://physlets.org/tracker/). The center of the microrobots was tracked automatically or manually to determine frame-by-frame positions. The displacement or coordinates of the videos were extracted from the file for further analysis. The displacement was recorded to calculate the velocity of the microrobots. The coordinates were used to show the movement trajectory.

### Statistical analysis

All statistical analyses of the experimental data were performed using Origin 2023b (OriginLab Co., Northampton, MA) and Prism 6 (GraphPad Inc.) software. Unless indicated otherwise, the experimental data are represented as the means ± SD.

## Supplementary Materials

### The PDF file includes:

Sections S1 to S5 Figs. S1 to S26 Table S1 Legends for movies S1 to S14 References
