# Supplementary material for: Permanent magnetic droplet–derived microrobots
Source: Sci Adv. 2025 Jul 9;11(28):eadw3172. doi: 10.1126/sciadv.adw3172 (PMC12239960; doi:10.1126/sciadv.adw3172)
Supplement: Supplementary file 1 — Sections S1 to S5 Figs. S1 to S26 Table S1 Legends for movies S1 to S14 References [file sciadv.adw3172_sm.pdf]

Science Advances
AAAS

Supplementary Materials for

Permanent magnetic droplet–derived microrobots

Yuanxiong Cao *et al.*

Corresponding author: Molly M. Stevens, molly.stevens@dpag.ox.ac.uk; Sharon C. Glotzer, sglotzer@umich.edu

Sci. Adv. **11**, eadw3172 (2025)
DOI: 10.1126/sciadv.adw3172

The PDF file includes:

Sections S1 to S5
Figs. S1 to S26
Table S1
Legends for movies S1 to S14
References

Other Supplementary Material for this manuscript includes the following:

Movies S1 to S14

Section S1. Fabrication of PMDMs

The formation of PMDMs is primarily influenced by the gravitational settling of the denser NdFeB microparticles. Given the relatively lower viscosity of the hydrogel compared to the hard-magnetic NdFeB, the microparticles tend to precipitate at the base of the hydrogel phase. To gain a clearer understanding of this sedimentation process, we used a sample of 80% (v/v) PEGDA hydrogel infused with 25% (w/v) NdFeB microparticles. As shown in **Fig. S1**, the hydrogel effectively retained the NdFeB microparticles with minimal noticeable sedimentation in the initial 3 min. This period aligns well with the droplet generation duration in the microfluidic process which allows for the production of approximately 900 PMDMs. Subsequently, a steady settling of the microparticles was observed, reaching an equilibrium after about 15 min. Based on this observation, the droplets were aerated for a minimum of 15 min to facilitate the complete settling of the microparticles and form the PMDMs.

To further evaluate the influence of NdFeB microparticles concentration on the sedimentation height h profile, we fabricated PMDMs with five different NdFeB concentrations: 5% (w/v), 25% (w/v), 50% (w/v), 75% (w/v), and 100% (w/v). As shown in **Fig. S3**, sedimentation height h increased with particle concentration. However, high concentrations also resulted in broader and less uniform distributions. Quantitative analysis based on over 40 PMDMs per condition revealed the following average sedimentation heights and standard deviations: 87.02 μm ± 15.53 μm (5% w/v NdFeB), 123.44 μm ± 21.46 μm (25% w/v NdFeB), 148.59 μm ± 21.19 μm (50% w/v NdFeB), 187.49 μm ± 39.98 μm (75% w/v NdFeB), 230.32 μm ± 41.94 μm (100% w/v NdFeB).

Among them, the 25% (w/v) NdFeB microparticles provided an optimal balance between magnetic strength and sedimentation uniformity, making this formulation ideal for all PMDM fabrication throughout the study unless notified.

We also investigated the magnetic properties of PMDMs with varying microparticle concentrations. As shown in **Fig. S4**, both remanent and saturated magnetization increased with increasing NdFeB content, supporting enhanced actuation performance at higher particle loadings.

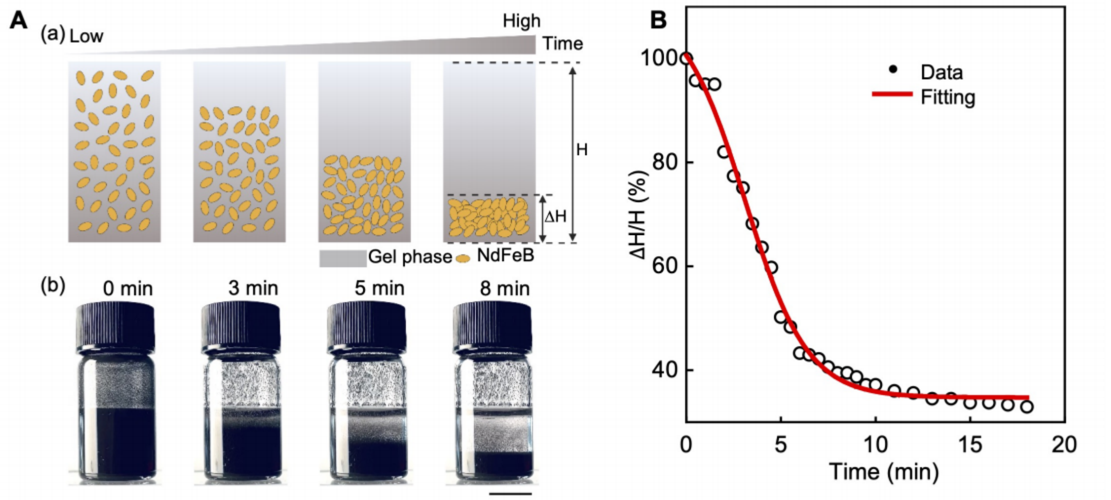

Fig. S1. Gravitational sedimentation induced Janus formation. (**A**) Schematic illustration (a) and time-lapsed snapshots (b) illustrating the phase separation during the sedimentation process. A Janus phase is formed within the ferromagnetic composite hydrogel ink induced by gravitational sedimentation, resulting in two distinct magnetic and non-magnetic phases within the ferromagnetic hydrogel. Scale bar, 1 cm. (**B**) Normalized sedimentation height over time.

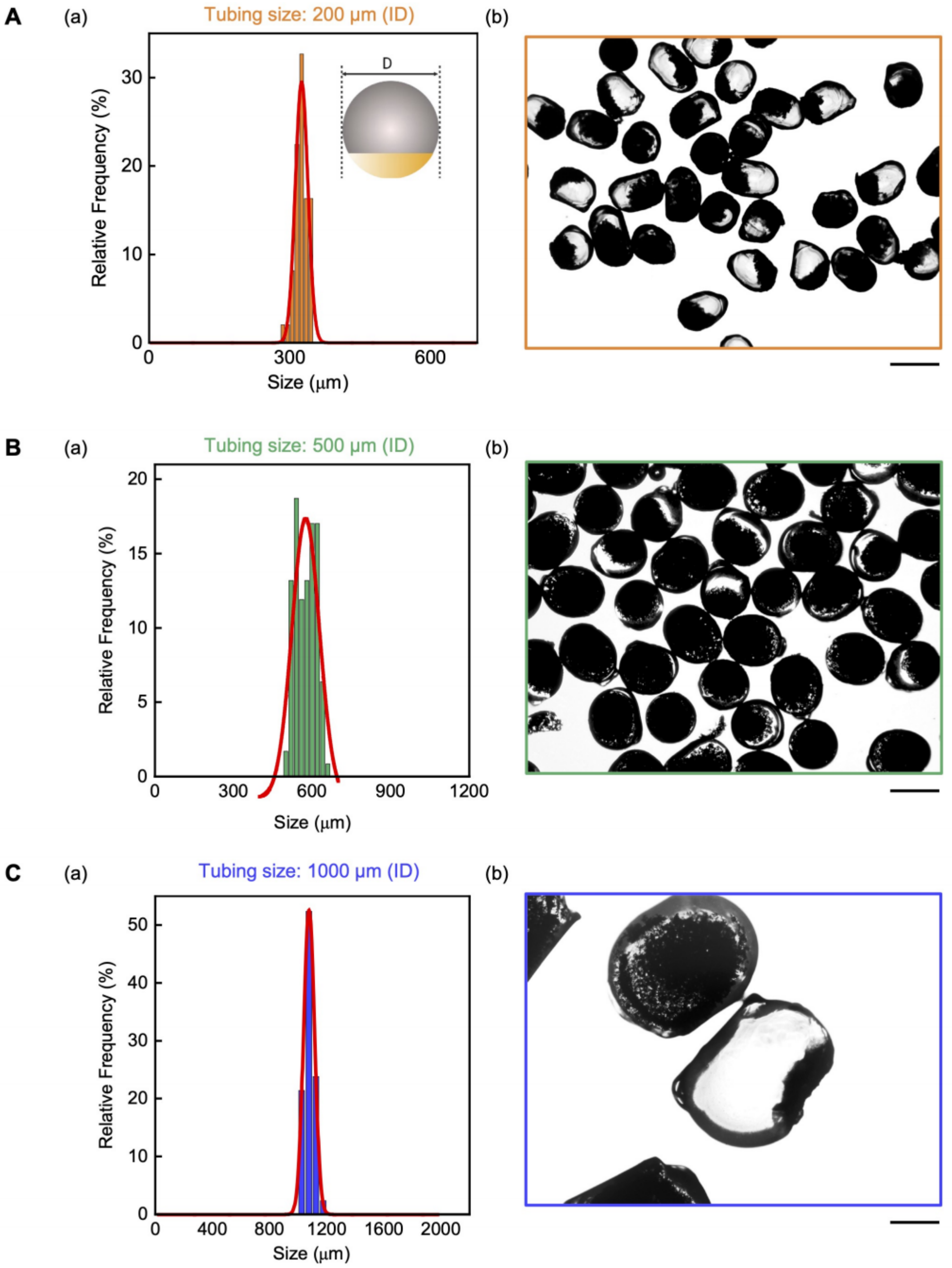

Fig. S2. Effect of tubing size on PMDMs size distribution. (**A-C**) (a-b) Size distribution (a) and microscopy image (b) of PMDMs produced using 200 µm (**A**), 500 µm (**B**), 1000 µm (**C**) internal diameter (ID) tubing. (n = 49 for **A**, n = 235 for **B**, n = 42 for **C**). Scale bars: 500 µm.

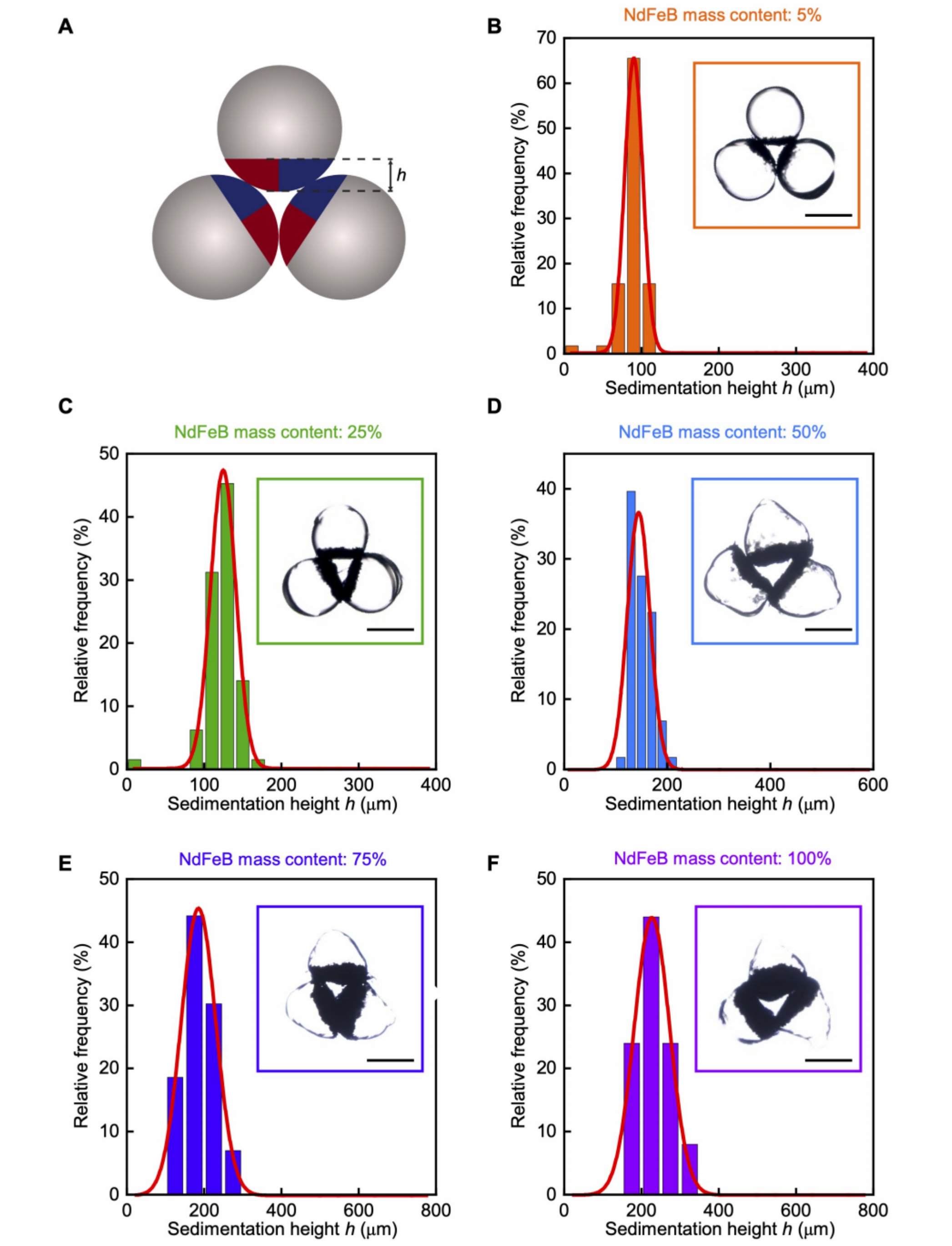

Fig. S3. Analysis of NdFeB microparticles sedimentation height h in PMDMs at varying concentrations. (**A**) Schematic illustration showing the definition of sedimentation height (h) in a PMDM cluster. (**B–F**) Histograms showing the frequency distribution of sedimentation height h for PMDMs fabricated with different NdFeB mass contents: (**B**) 5% (w/v), (**C**) 25% (w/v), (**D**) 50% (w/v), (**E**) 75% (w/v), and (**F**) 100% (w/v). Each histogram was generated from >40 individual PMDMs. Insets show representative images of the magnetic regions within the PMDMs at each condition. Scale bars, 500 μm.

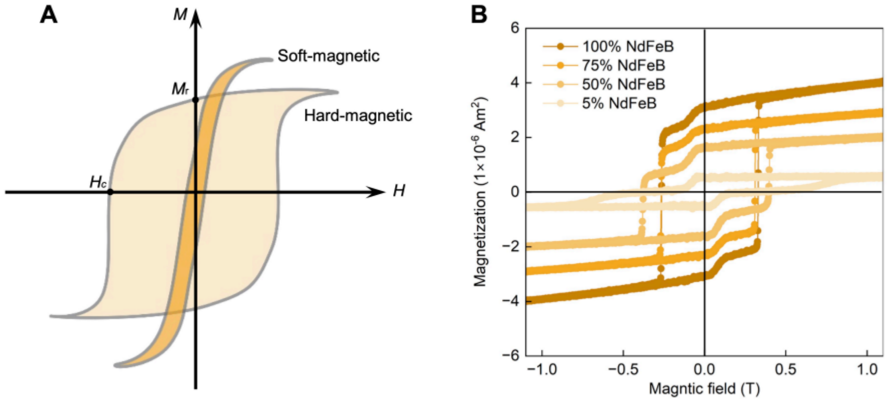

Fig. S4. Magnetic properties of ferromagnetic composite hydrogel ink. (**A**) Schematic of the magnetization curves with magnetic hysteresis loops of soft-magnetic and hard-magnetic materials. Soft-magnetic materials possess high saturation magnetization and low coercivity H_c, indicating they can be easily magnetized in the presence of a magnetic field and quickly lose their magnetization once the field is removed. Hard-magnetic materials exhibit high coercivity H_c, enabling them to retain their magnetic properties even in the absence of an applied magnetic field. (**B**) Magnetic hysteresis loop of single microrobot with varied NdFeB microparticle content. The microrobots, comprising 80% (v/v) PEGDA and varying concentrations of NdFeB microparticles, including 5% (w/v), 50% (w/v), 75% (w/v), and 100% (w/v), were characterized using a vibrating sample magnetometer.

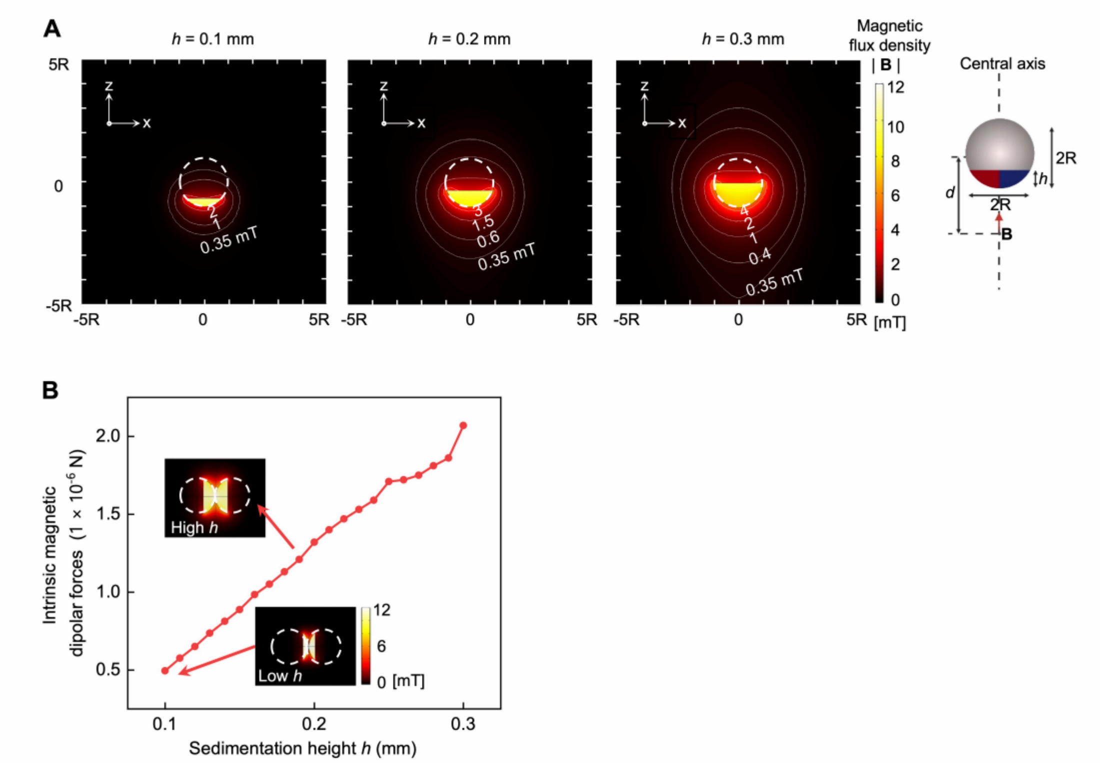

Fig. S5. Analysis of the PMDM magnetic field distribution and intrinsic magnetic dipolar forces by finite element simulation. (**A**) Simulation analysis of magnetic flux density distribution surrounding a single PMDM, with varying sedimentation heights h of 0.1 mm, 0.2 mm, and 0.3 mm. (**B**) The intrinsic magnetic dipolar forces between two assembled PMDMs with varying sedimentation heights h. Low and high h denote 0.1 mm and 0.3 mm, respectively. The white dotted circle represents the boundary of the PMDM.

Section S2. Dynamic self-assembly of PMDMs

The assembly process in the experiments was tracked using the commercially available software Tracker version 6.1.3 (https://physlets.org/tracker/). The microrobots can be tracked automatically or manually to label the moving position.

In the case of PMDM dimer assembly, the trajectory of each PMDM appears symmetrical about the centroid during the approach, as shown in **Fig. S6**. This behavior indicates that the forces acting on the PMDMs, such as the external magnetic field interaction force and the hydrodynamic force induced by the fluid environment, are evenly distributed.

However, the assembly dynamics shift when dealing with multiple PMDMs. For microrobots possessing a lower sedimentation height h, the trajectories of individual PMDMs appear arbitrary. This random behavior likely stems from the weaker electromagnetic interactions between the PMDMs, making it challenging for them to coalesce into a unified chain. Conversely, for PMDMs with a greater sedimentation height h, the trajectories are more predictable. These PMDMs tend to move from the periphery towards the center, culminating in the formation of a PMDM chain.

Considering the PMDM chain fragments, it can essentially be perceived as possessing axial magnetization. Consequently, each end of an individual PMDM chain is characterized as either the North (N) or South (S) pole. Upon the application of a rotating magnetic field, the N pole of one PMDM chain fragment attracts the S pole of another, facilitating the creation of an elongated chain.

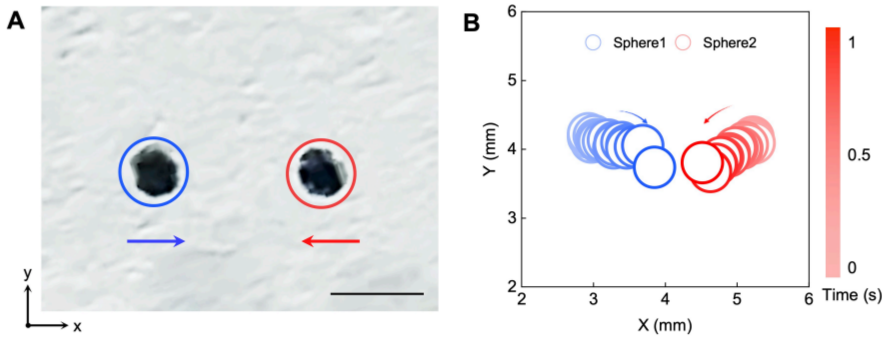

Fig. S6. Temporal trajectory analysis of a PMDM dimer assembly process. (A) Snapshot illustrating the convergence of two individual PMDMs, represented by solid circles in red and blue. Scale bar: 1 mm. (**B**) Temporal evolution of the two approaching PMDMs, depicted by overlaying their identified outlines (depicted as solid lines) captured at different time frames. The color shading indicates the temporal sequence.

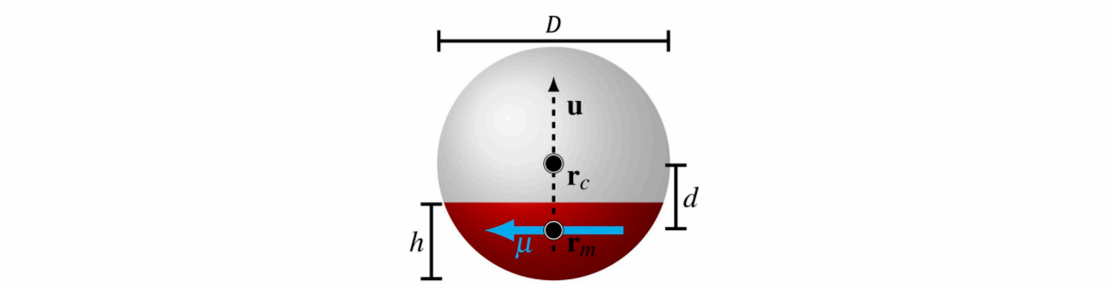

Fig. S7. Schematic illustration of the model of a single PMDM used in MD simulation. r_c and r_m represent the geometrical center position and the center of mass of the magnetic part, respectively. u is the normalized orientation vector. h, d, and σ indicate the sedimentation height of NdFeB microparticles, the distance to the geometrical center and the diameter of the PMDM, respectively.

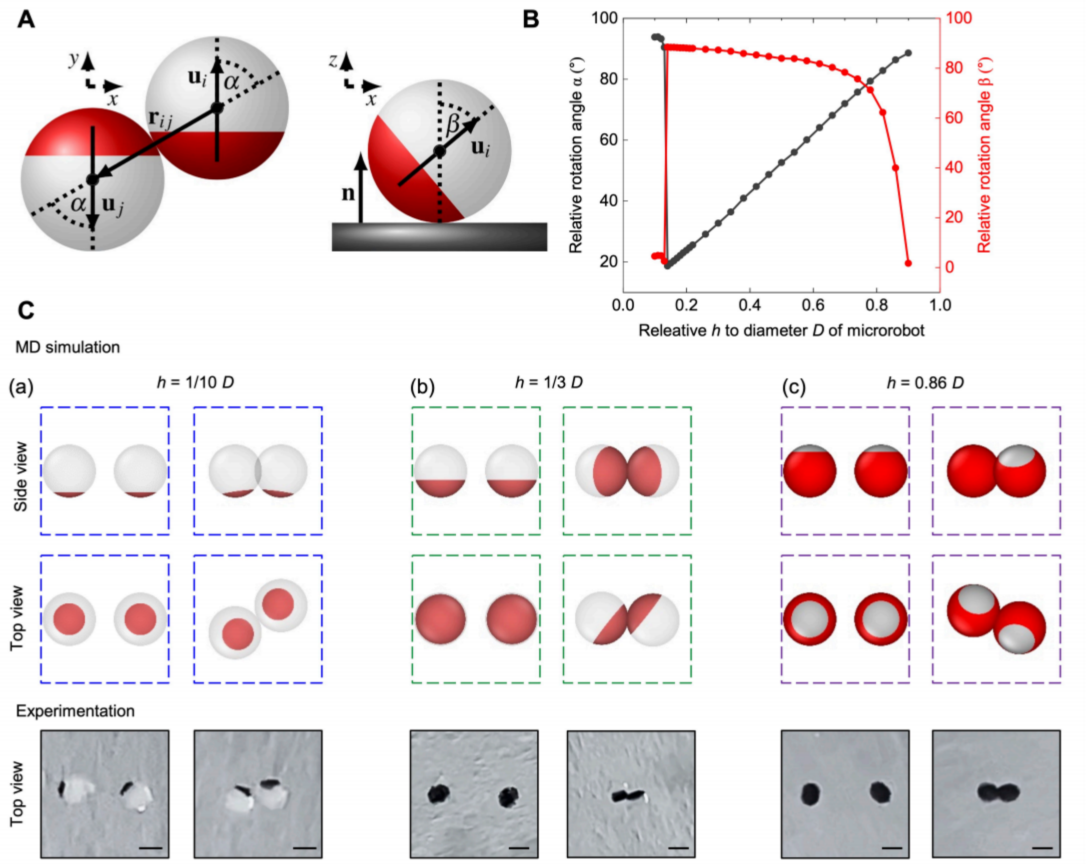

Figure S8. MD simulation predicts the self-assembly of PMDMs in the experiment. (**A**) The angle α depicts the relative rotational angle between the dimer PMDMs and the angle β represents the relative inclination angle of PMDMs to the interface. (**B**) Plot showing the variation of α and β with the relative $h{:}D$ ratio of the PMDM. (**C**) Snapshots showing experimental data and MD simulation at different $h{:}D$ ratios, including 1/10 (a), 1/3 (b), and 0.86 (c). Scale bars, 500 µm.

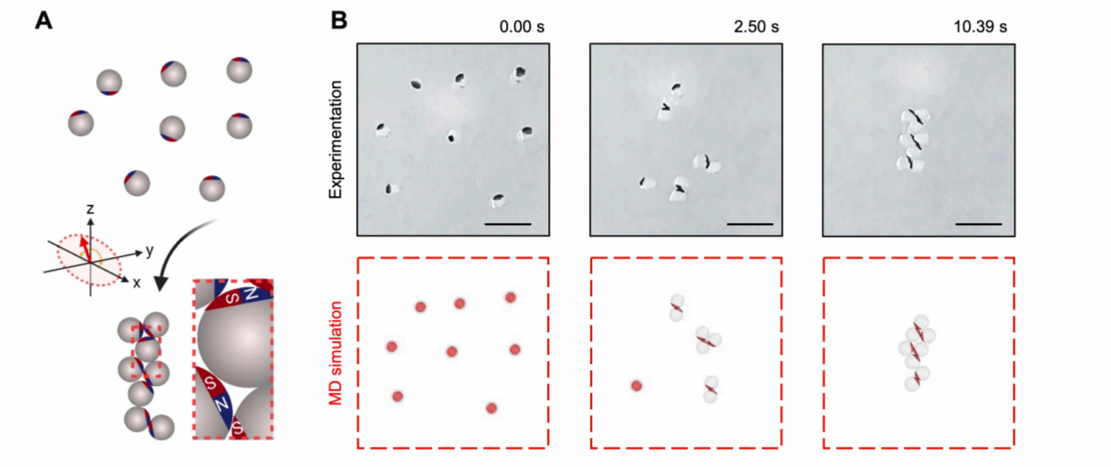

Fig. S9. Assembly of PMDMs with low sedimentation height *h*. (**A**) Schematic showing the assembly of PMDMs with low sedimentation height *h*. (**B**) Snapshots showing the experiment and MD simulation of the assembly process. Scale bar, 2 mm.

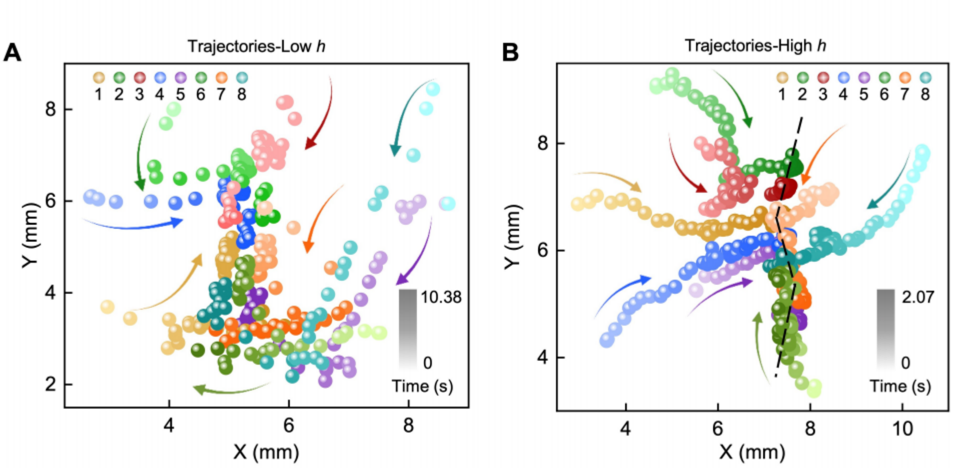

Fig. S10. Temporal trajectory analysis of the assembly process of PMDMs in experiment with different sedimentation heights. The assembly process is based on PMDMs with low sedimentation heights *h* (**A**) and high sedimentation heights *h* (**B**). The color of the PMDM is modified in accordance with the temporal sequence.

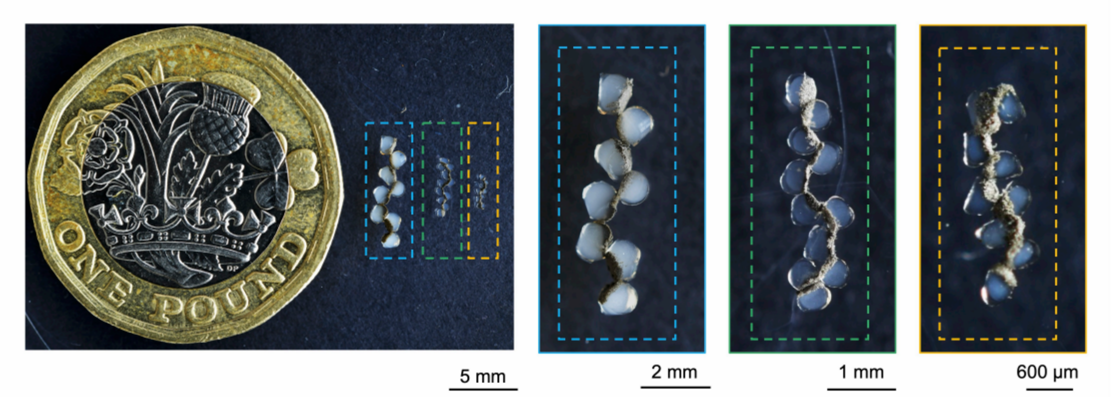

Fig. S11. Size comparison of the assembled PMDM chains. The assembled PMDM chains were produced using 200 µm, 500 µm and 1000 µm ID tubing.

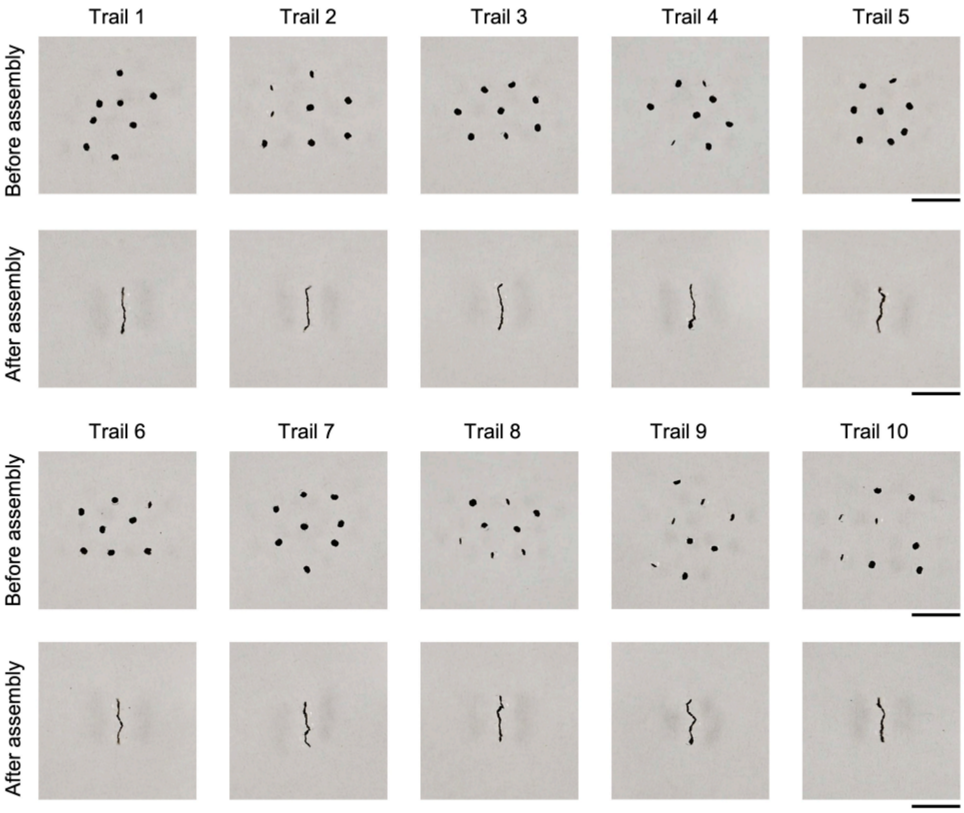

Fig. S12. Reproducibility of PMDM chain assembly across 10 independent trials. Optical images of eight PMDMs before magnetic actuation in 10 separate trials before and after assembly into PMDM chains by application of a magnetic field. Scale bars: 5 mm.

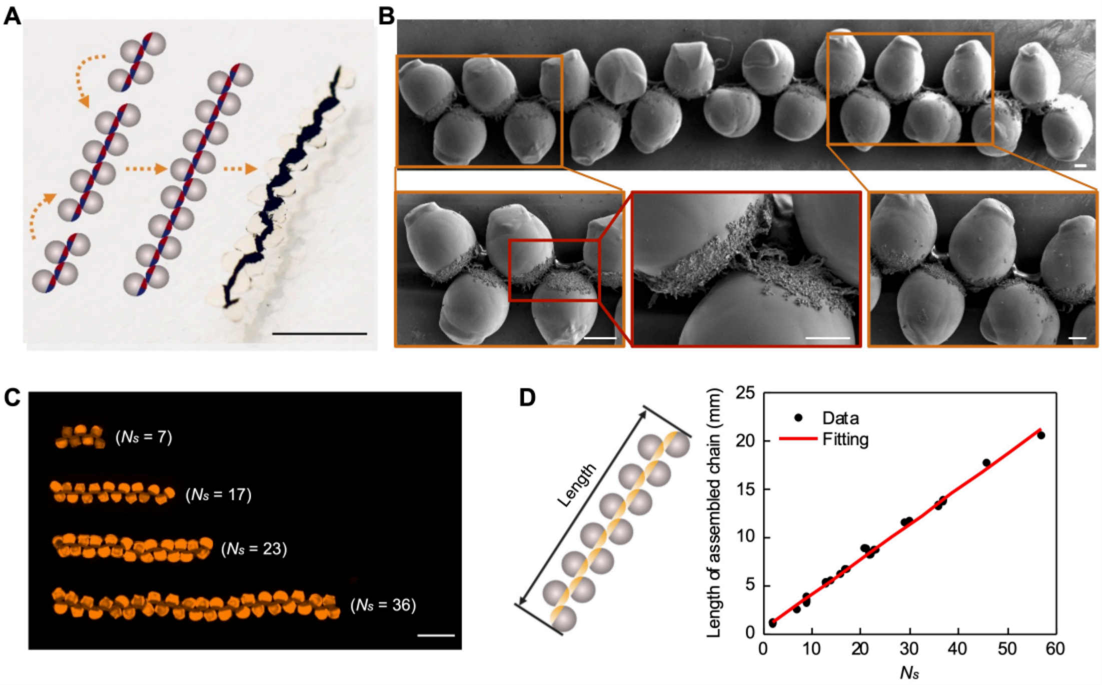

Fig. S13. Characterization of assembled PMDM chains. (**A**) An elongated zigzag PMDM chain assembled from shorter chain fragments. The image is taken in the absence of an external magnetic field. Scale bar, 2 mm. (**B**) SEM characterization of an elongated zigzag PMDM chain. The images provide intricate structural details of the assembled PMDMs, explicitly revealing the spatial arrangement of NdFeB microparticles within the composite chain. Scale bar: 100 µm. (**C**) Fluorescent images of PMDM chains comprising different numbers of PMDMs (N_S = 7, 17, 23, 36). Each PMDM was loaded with 1 µL/mL FluoSphere carboxylate (diameter = 0.2 µm, orange). Scale bar, 1 mm. (**D**) The length of assembled PMDM chains is linearly proportional to the number N_s of assembled PMDMs.

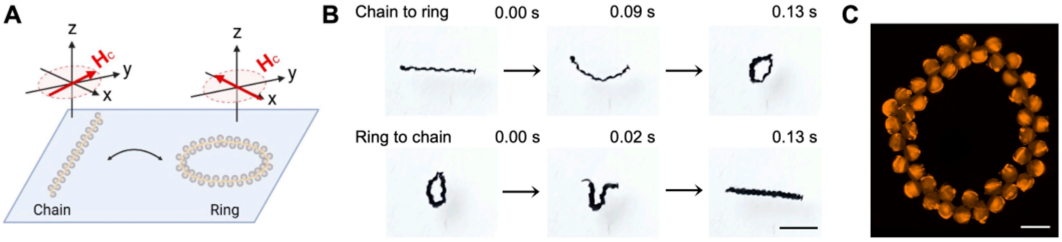

Fig. S14. Reversible transformation between PMDM chain and ring structures. (**A**) Schematic depiction illustrating the reversible transition between chain and ring configuration induced by altering the direction of the applied horizontal magnetic field. (**B**) Snapshots captured at specified time intervals, showcasing the dynamic transformation between the chain and ring configurations. Scale bar, 3 mm. (**C**) Fluorescent image of a fully assembled PMDM ring comprising of 44 individual PMDMs. Each PMDM was loaded with 1 µL/mL FluoSphere carboxylate (D = 0.2 µm, orange). Scale bar, 1 mm.

Section S3. Multimodal locomotion of PMDM chains:

(1) Walking

When applying a rotating magnetic field in the vertical plane, the PMDM chain rotates up due to the induced magnetic torque (**Fig. 3C**). The rotating magnetic field can be expressed as:

$$\vec{B}\,(f,t) = B[cos\,cos\,(2\pi f t)\,u + v] \tag{S1}$$

where B is the magnitude of the magnetic field, f is the rotating frequency, u and v represent the base vectors in the rotating plane. Thus, the magnetic torque on the PMDM chain can be described as follows,

$$T_m = V_m|M||B|sin(2\pi f t - \varphi) \tag{S2}$$

where phase angle φ is the sum up of rotational angle θ and the angle of magnetic alignment β. Since the magnetic field continuously rotates, it generates a driving force that propels the PMDM chain forward. The force equation on the robot is given by,

$$F_{dri} - F_h - F_f = m\dot{V}_{walk} \tag{S3}$$

where F_{dri} is the driving force acted on the robot, F_h is the hydrodynamic force and F_f is the friction, V_{walk} is the walking speed and m is the mass of the assembled PMDM chain. V_{walk} can be described as follows,

$$V_{walk} = \dot{\theta}\frac{L}{2} \tag{S4}$$

$$\dot{V}_{walk} = \ddot{\theta}\frac{L}{2} \tag{S5}$$

The motion equation about the rotation axis of the PMDM can be expressed as follows,

$$T_m - C_R\dot{\theta} - F\frac{L}{2} = J\ddot{\theta} \tag{S6}$$

where C_R is the rotational damping coefficient, F is induced from hydrodynamic force and friction, J is the moment of inertia. Ignore the slipping, the motion equation can be described as follows,

$$V_m|M||B|\,sin\,sin\,(2\pi f t - \varphi) = J\ddot{\theta} + C_R\dot{\theta} \tag{S7}$$

J can be ignored since the size of the robot is relatively small, then simplifying equation (3.7) to equation (3.8),

$$\dot{\theta} = \frac{V_m|M||B|sinsin\,(2\pi f t-\varphi)}{C_R} = \frac{V_m|M||B|sinsin\,(2\pi f t-\theta-\beta)}{C_R} \tag{S8}$$

When $sin\,(2\pi f t - \theta - \beta) = 0$, it reaches the step out frequency. Usually, in the walking motion, the frequency of the rotating magnetic field is relatively lower than the step out frequency. Once the robot reaches its steady-state speed, there is a constant lag angle between the $\vec{B}$ and the rotational angle θ. It can be expressed as $2\pi f t - \theta = A_{lag}$, and further deduced it into equation (3.9) by differentiating this equation with respect to t,

$$\dot{\theta} = 2\pi f \tag{S9}$$

Therefore, the walking speed can be determined using equation (3.10),

$$V_{walk} = 2\pi f\frac{L}{2} \tag{S10}$$

This indicates that the walking speed is linearly proportional to the frequency of the rotating magnetic field f, and the length of the PMDM chain L, which is verified with the experimental data (**Fig. 3C c**). At a magnetic field strength of 4 mT and a frequency of 1 Hz, the PMDM chain exhibited vertical walking behavior, as shown in **Fig. 3C b**.

(2) Crawling

When a vertically oscillating magnetic field is applied, the PMDM chain can crawl on the surface (**Fig. 3D**). The oscillating magnetic field can be expressed as:

$$\vec{B}(f,t) = B[|cos(2\pi f t)|n + sin(2\pi f t)u] \tag{S11}$$

where B is the magnitude of the magnetic field, f is the rotating frequency, n and u represent the base vectors in the oscillating plane. Thus, the dynamic force on the PMDM chain can be expressed as:

$$\vec{T}_m = V_m\vec{M} \times \vec{B} \tag{S12}$$

$$m\ddot{y} = F_f - F_h - F_m cos\left(\frac{\pi}{2} - \theta_\alpha\right) \quad \text{(S13)}$$

$$m\ddot{z} = N + F_m sin\left(\frac{\pi}{2} - \theta_\alpha\right) - mg \quad \text{(S14)}$$

$$J\ddot{\theta}_\alpha = T_m - T_h + F_f \frac{L}{2} sin\theta_\alpha - F_h \frac{L}{2} cos\theta_\alpha \quad \text{(S15)}$$

where θ_α is the pitch angle, F_f is the friction, which acts as the driving force on the robot, F_h is the hydrodynamic force, F_m is the magnetic force obtained by the magnetic torque T_m on the robot, and J is the polar moment of the PMDM chain.

(3) Swinging

When we apply an oscillating magnetic field in the vertical plane, the PMDM chain swings on the surface (**Fig. 3E**). The oscillating magnetic field can be expressed as:

$$\vec{B}(f,t) = B[|cos(2\pi ft)|n - sin(2\pi ft)u] \quad \text{(S16)}$$

where B is the magnitude of the magnetic field, f is the rotating frequency, n and u represent the base vectors in the oscillating plane. Similar to the crawling motion, the dynamic force on the PMDM chain can be expressed as:

$$m\ddot{y} = F_m cos\theta_s - F_f - F_h \quad \text{(S17)}$$

$$m\ddot{z} = N + F_m sin\theta_s - mg \quad \text{(S18)}$$

$$J\ddot{\theta}_\alpha = T_m - T_h - F_f \frac{L}{2} cos\theta_s - F_h \frac{L}{2} sin\theta_s \quad \text{(S19)}$$

where θ_s is the angle between the advancing direction of the PMDM chain and y-axis, F_f is the friction, F_h is the hydrodynamic force, F_m is the magnetic force obtained by the magnetic torque T_m on the robot, and J is the polar moment of the PMDM chain.

(4) PMDM chains ascent and descent stairs

The motion sequence of a PMDM chain climbing up the stairs can be described by several distinct stages. Initially, the front part of the chain remained stationary, serving as the fulcrum. When a rotating magnetic field was applied in the vertical plane, the centroid and the rear part of the chain rotated upwards and attached to the upper step upon gaining traction. Subsequently, the rear part of the chain became the fulcrum, allowing the front part of the chain to rotate upwards and attach to the next upper stair. This alternating fulcrum mechanism enabled the PMDM to climb. This was demonstrated with a PMDM of body length = 5 mm that successfully climbed up and down stairs continuously (2 mm width and 2 mm height for each step).

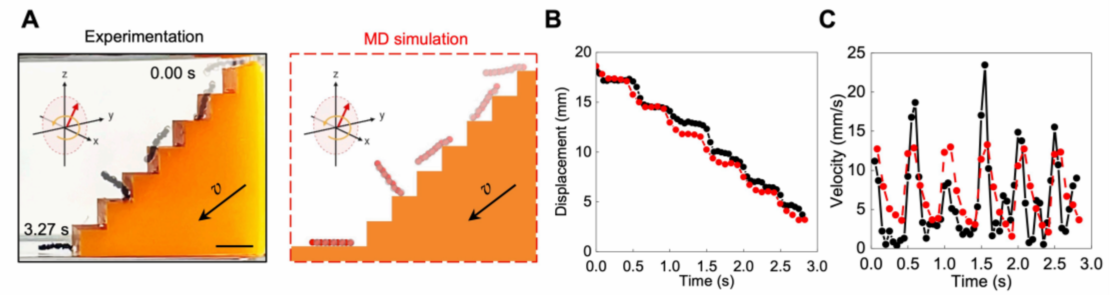

Fig. S15. The assembled PMDM chain descends the stairs. (**A**) Assembled PMDM chain descending stairs. Scale bar, 2 mm. (**B-C**) Comparison between experimental and MD simulation results of the displacement (**B**) and the velocity profile (**C**) of the centroid of the chain over time.

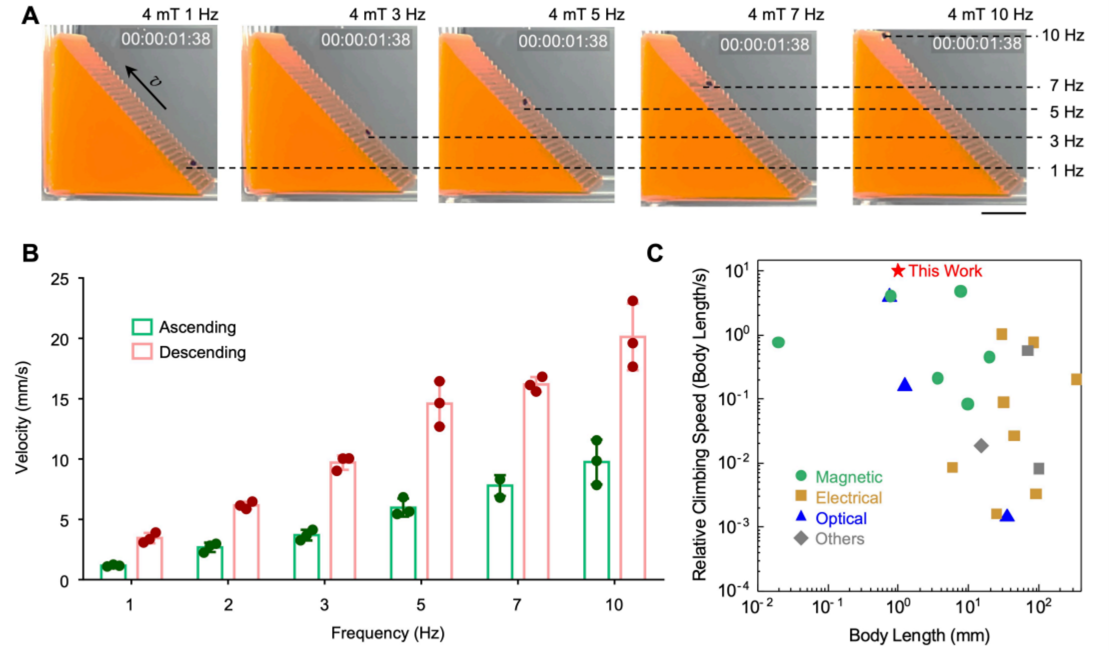

Fig. S16. PMDM dimers ascending and descending stairs. (**A**) PMDM dimers climbing on stairs (0.4 mm width and 0.4 mm step height) at different rotating magnetic field frequencies. The PMDMs start climbing from the same point and are exposed to the same magnetic field strength (4 mT). Scale bar: 5 mm. (**B**) Average ascent and descent velocity of PMDM dimers climbing stairs at different rotating magnetic field frequencies. The values are represented as mean ± SD (n = 3). (**C**) Comparison of relative climbing speed in relation to body length for various actuation mechanisms reported in the literature: magnetic (*50-55*), optical (*56-58*), electrical (*59-66*), and other (*67-69*).

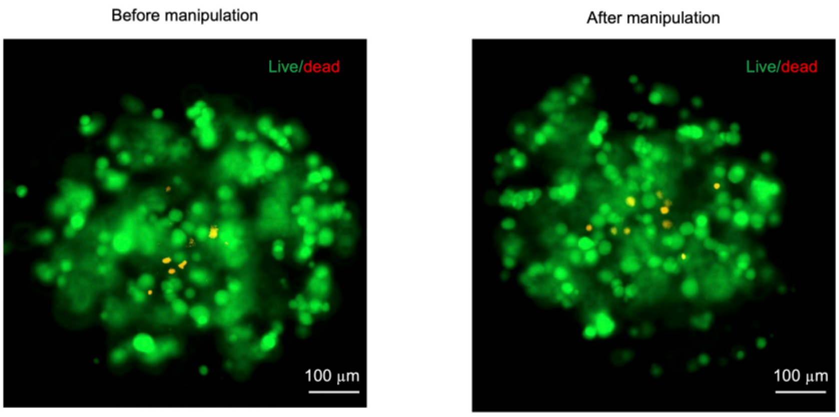

Fig. S17. Cell viability of hMSCs-laden Matrigel microspheres before and after manipulation. Scale bars, 100 μm.

Section S4. Disassembly and assembly of PMDMs

When a rotating magnetic field is applied, the PMDM chains can be assembled or disassembled. The Mason number R_T estimates the stability of a rotating chain, which is always defined as the ratio of the hydrodynamic force F_h to the electromagnetic force F_m:

$$R_T = \frac{2\pi f \eta}{\mu_0 \chi_p^2 H_0^2} \tag{S20}$$

A lower Mason number value indicates that the magnetic influence outweighs the viscous one, enabling particles to either form a chain or maintain their magnetized state. Conversely, a larger Mason number implies the opposite. The Mason number R_T of a rotating particle chain can be further defined as (*70*):

$$R_T = \frac{32\pi f \eta}{\mu_0 \chi_p^2 H_0^2} \frac{N^3}{(N-1)\left(ln\left(\frac{N}{2}\right)+\frac{2.4}{N}\right)} \tag{S21}$$

Here, f is the frequency of the magnetic field in rotation, and *N* stands for the number of PMDMs that compose the chain. Given the chain's length as *L*=4*NR*, and setting R_T=1, we can deduce the connection between the frequency *f* of the magnetic field and the chain length *L*:

$$f = \frac{8\mu_0 \chi_p^2 H_0^2}{\pi f \eta} \frac{(L-4R)\left(ln\left(\frac{L}{8R}\right)+\frac{9.6R}{L}\right)}{L^3} \tag{S22}$$

Where $L \gg R$, so it can be concluded that the rotating magnetic frequency f is inversely proportional to its length L. This implies that as the frequency escalates, R_T increases leading to the chain breaking apart. On the other hand, as the frequency decreases, a rise in R_T results in the chain's formation.

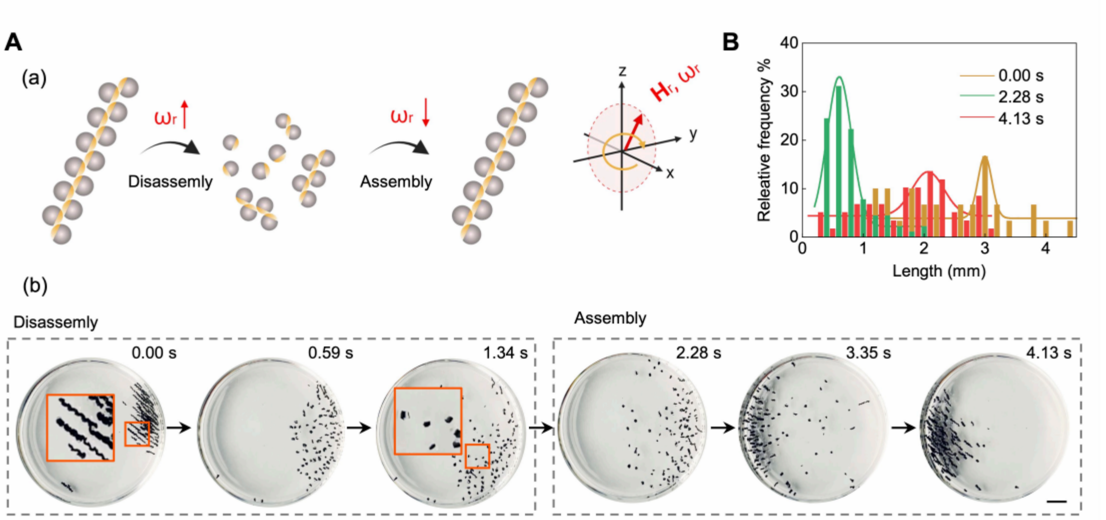

Fig. S18. Reconfigurable transformation of assembled PMDM chains. (**A**) Schematic illustration (a) and sequential snapshots (b) demonstrating the splitting and merging of swarm PMDM chains. Swarm PMDM chains split and merge when the frequency of the vertical rotating magnetic field is increased or decreased, respectively. Scale bar: 5 mm. (**B**) Chain length distribution analysis during dynamic splitting and merging process. The corresponding distribution histograms illustrate the transition of the assembled chains throughout the splitting and merging processes.

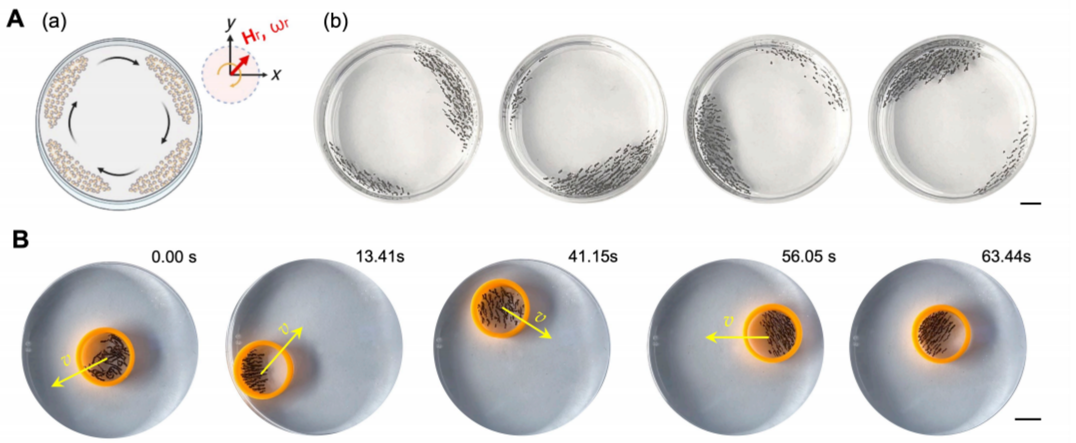

Fig. S19. Manipulation of swarm PMDMs. (**A**) Schematic illustration (a) and sequential snapshots (b) demonstrating that swarm PMDM chains move in a circular path by applying a rotating magnetic field at the *xy* plane. (**B**) Swarm PMDM chains transporting a ring (ID = 14 mm, OD = 16 mm, height = 8 mm, 0.45 g). Scale bars: 5 mm.

Section S5. Cell and drug encapsulation using PMDMs

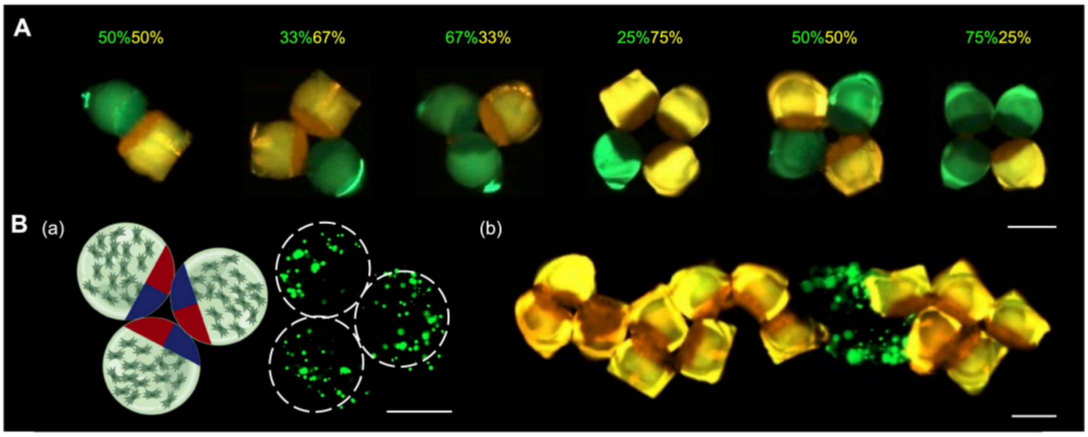

Fig. S20. Co-encapsulation of drugs and cells. (A) Fluorescent images of assembled PMDM dimers, trimers, and tetramers each loaded with different proportions of dual FluoSphere carboxylates. The PMDM is composed of 80% (v/v) PEGDA matrix with 25% (w/v) NdFeB microparticles. Scale bar, 500 µm. (**B**) (a-b) Fluorescent images demonstrating the versatility of PMDMs to carry cells only (a) and both drugs and cells (b). hMSCs stained by CellTracker™ Green CMFDA are encapsulated within a complex of 1% (w/v) alginate-5% (w/v) gelatin with 25% (w/v) NdFeB microparticles. A model drug is represented by fluorospheres with a diameter of 0.2 µm and a concentration of 20 µL/mL, encased within an 80% (v/v) PEGDA matrix with 25% (w/v) NdFeB microparticles. Scale bars: 500 µm.

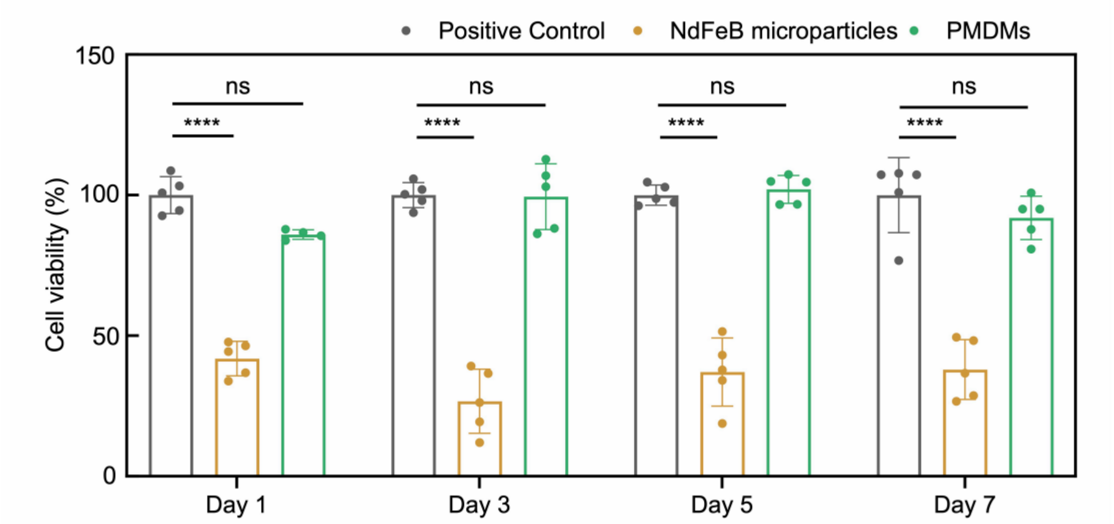

Fig. S21. Cell viability assay. Cell viability of HUVECs co-cultured with NdFeB microparticles or PMDMs. The PMDMs consist of 80% (v/v) PEGDA containing 25% (w/v) NdFeB microparticles. The positive control group is cultured at normal conditions. All values are represented as mean ± SD (n ⩾ 4). Statistical difference determined by two-way ANOVA; ****$p < 0.0001$.

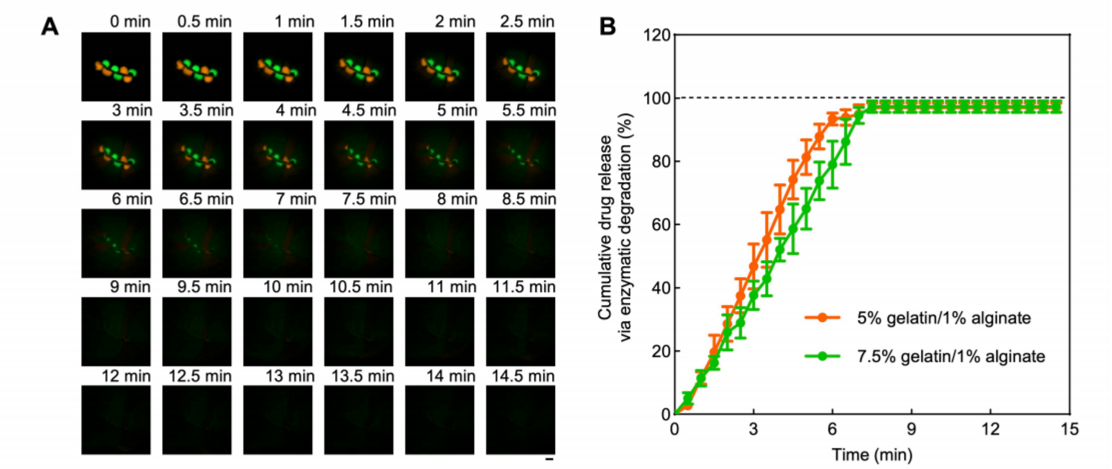

Fig. S22. Release kinetics of encapsulated fluorescent microspheres from PMDM chains during enzymatic degradation. (**A**) Time-lapse fluorescence images showing the dual release of encapsulated fluorescent microspheres from PMDM chains under enzymatic degradation. Green fluorescence: D = 0.2 μm, 20 µL/mL, dissolved within a composite matrix that includes 1% (w/v) alginate-7.5% (w/v) gelatin with 25% (w/v) NdFeB microparticles. Yellow fluorescence: D = 0.2 μm, 20 µL/mL, dissolved within a composite matrix that includes 1% (w/v) alginate-5% (w/v) gelatin with 25% (w/v) NdFeB microparticles. Scale bar, 500 µm. (**B**) Quantification of cumulative drug release as a function of time, calculated by measuring the fluorescence area in panel A. Data represent mean ± SD (n = 4).

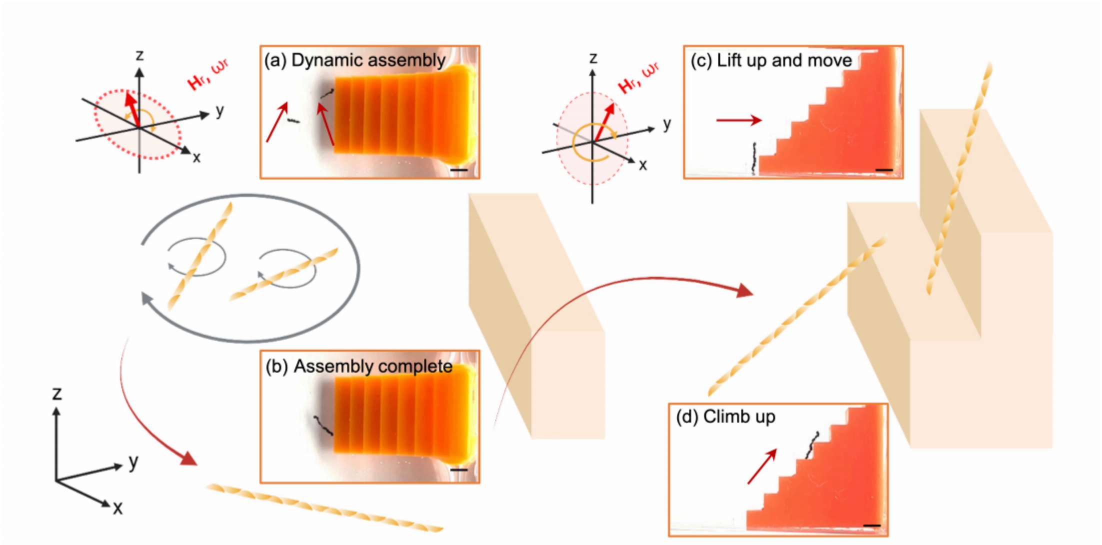

Fig. S23. Adaptive locomotion of degraded PMDM chain ascending and descending stairs. Degraded assembled PMDM chain fragments first assemble into an elongated PMDM chain and then climb up the stairs. The hydrogel phase has been fully degraded in the chains. Scale bars, 2 mm.

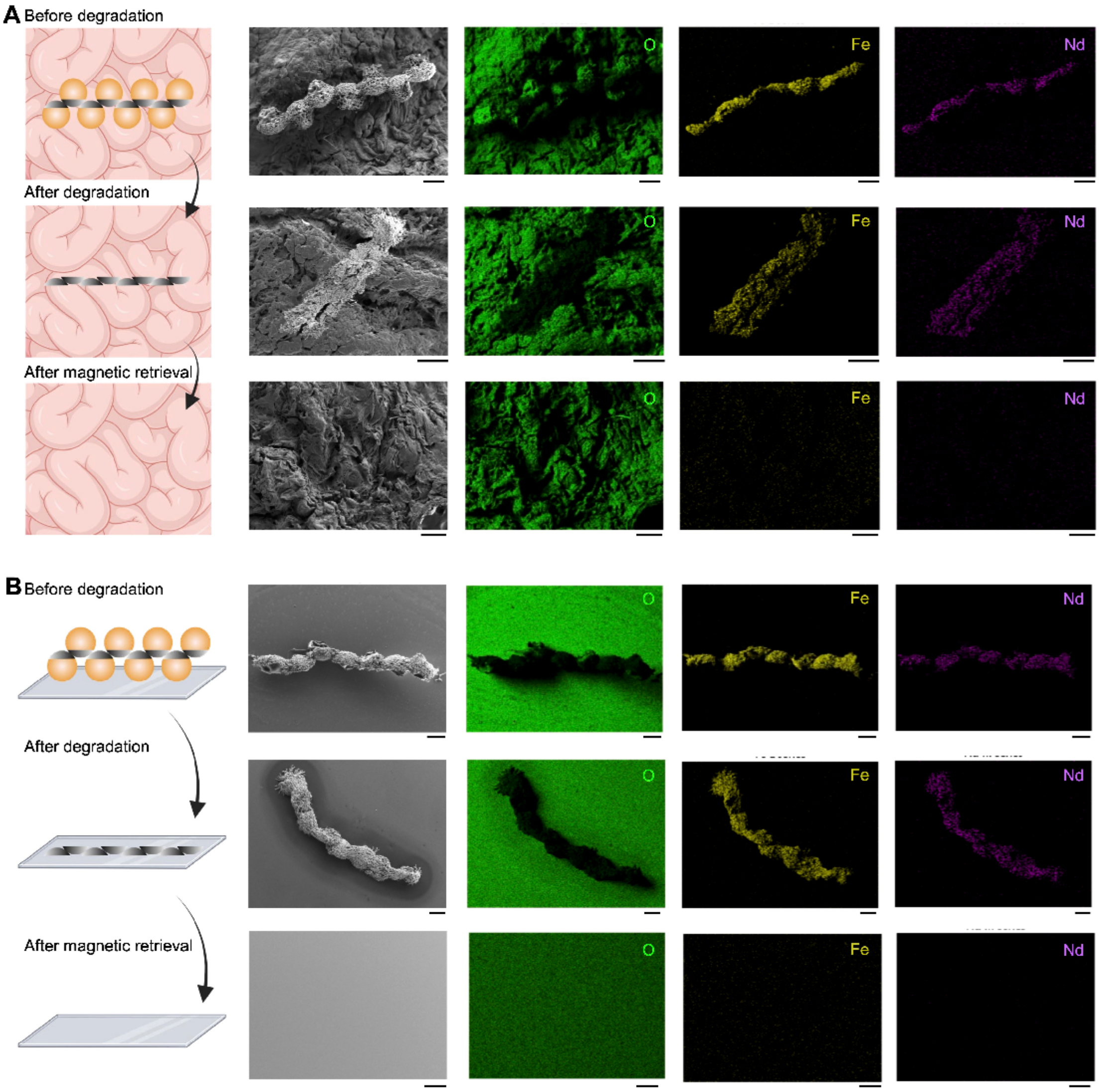

Fig. S24. Evaluation of magnetic particle retention and recovery after hydrogel degradation on different substrates. (A) Schematic illustration and corresponding experimental images (SEM images, EDS elemental maps for oxygen (green), iron (yellow), and neodymium (purple)) showing the behavior of PMDMs before degradation, after full enzymatic degradation, and after magnetic retrieval when placed on ex vivo porcine intestinal tissue **(B)** Same experimental setup as in (A), performed on a clean glass substrate. All scale bars: 200 μm.

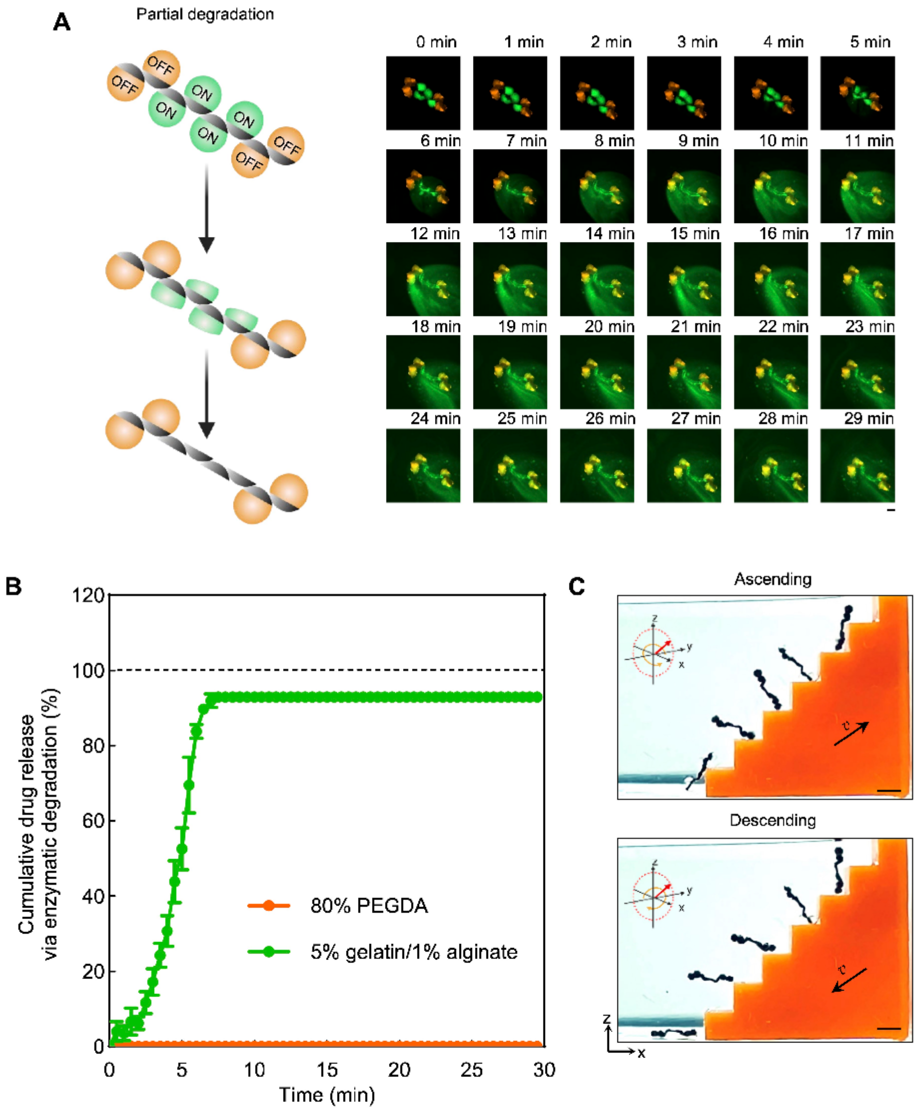

Fig. S25. Programmable sequential release and post-release locomotion behavior of PMDM chains. (A) Schematic and time-lapse fluorescence images illustrating sequential release behavior enabled by spatially patterned degradation. Scale bar, 500 µm. Green: D = 0.2 µm, 20 µL/mL, dissolved within a composite matrix that includes 1% (w/v) alginate-5% (w/v) gelatin with 25% (w/v) NdFeB microparticles. Yellow: D = 0.2 µm, 20 µL/mL, dissolved within a composite matrix that includes 80% (v/v) PEGDA with 25% (w/v) NdFeB microparticles. (**B**) Quantification of cumulative drug release from the two hydrogel formulations under enzymatic degradation. Data represents mean ± SD (n = 4). (**C**) Degraded chains ascent and descent stairs. Scale bars, 2 mm.

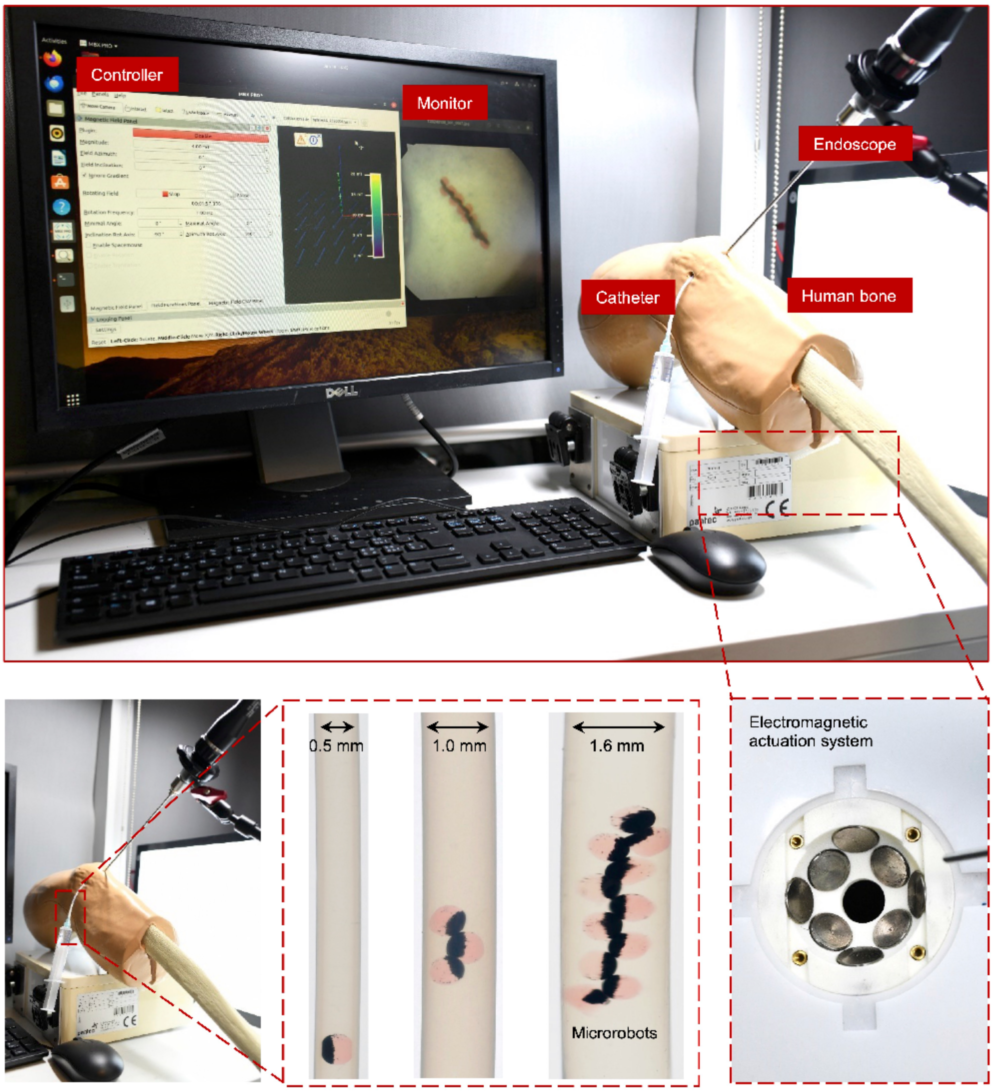

Fig. S26. Endoscope-assisted PMDMs delivery platform. The experimental setup used for navigating PMDMs within a human bone model consists of a controller, a monitor, an electromagnetic actuation system, a catheter loading PMDMs, an endoscope, and a human bone model. The PMDMs with different numbers can be loaded within different catheters with varied sizes, including 0.5 mm, 1 mm, and 2 mm.

Table S1. Data of relative speed versus body length of soft robots with different actuation methods

Actuation methods	Body length (mm)	Relative speed (BL/s)	Reference
**Magnetic**			
1	7.8	4.6*	(*50*)
2	0.8	3.9*	(*51*)
3	10	0.8*	(*52*)
4	0.02	0.75*	(*53*)
5	20	0.44*	(*54*)
6	3.7	0.2*	(*55*)
**Optical**			
1	0.75	3.95	(*56*)
2	1.25	0.16	(*57*)
3	35	0.0014*	(*58*)
**Electrical**			
1	30	1	(*59*)
2	85	0.75	(*60*)
3	350	0.2	(*61*)
4	32	0.087*	(*62*)
5	45	0.026	(*63*)
6	6	0.008*	(*64*)
7	91	0.0033	(*65*)
8	25	0.0016	(*66*)
**Others**			
1	70	0.56	(*67*)
2	15	0.0187	(*68*)
3	100	0.008	(*69*)
Magnetic	1	9.85	**This work**
*Estimated relative speed			

Supplementary Movies

Movie S1. PMDMs fabrication.

Movie S2. Oscillation Induced Assembly of PMDM Dimer.

Movie S3. Oscillation Induced Assembly of Eight PMDMs.

Movie S4. Oscillation Induced Assembly of PMDM Chain Fragments.

Movie S5. Multimodal Locomotion of the Assembled PMDM Chains.

Movie S6. Traversing Various Terrains.

Movie S7. Crossing Inclined Surfaces with Different Slopes.

Movie S8. Climbing Stairs with Different Rotating Magnetic Field Frequencies.

Movie S9. Manipulation of Assembled PMDM Chains.

Movie S10. Reconfigurable Transformation and Adaptive Locomotion of Assembled PMDM Chains.

Movie S11. Manipulation of Swarm PMDM Chains.

Movie S12. Assembly and Navigation on Porcine Intestine Surface ex vivo.

Movie S13. Controlled Drug Release Using Assembled PMDM Chains.

Movie S14. Endoscopy-assisted PMDMs Delivery Platform.

REFERENCES AND NOTES

1. H. Xie, M. Sun, X. Fan, Z. Lin, W. Chen, L. Wang, L. Dong, Q. He, Reconfigurable magnetic microrobot swarm: Multimode transformation, locomotion, and manipulation. *Sci. Robot.* **4**, eaav8006 (2019).

2. G. Gardi, S. Ceron, W. Wang, K. Petersen, M. Sitti, Microrobot collectives with reconfigurable morphologies, behaviors, and functions. *Nat. Commun.* **13**, 2239 (2022).

3. A. T. Liu, M. Hempel, J. F. Yang, A. M. Brooks, A. Pervan, V. B. Koman, G. Zhang, D. Kozawa, S. Yang, D. I. Goldman, M. Z. Miskin, A. W. Richa, D. Randall, T. D. Murphey, T. Palacios, M. S. Strano, Colloidal robotics. *Nat. Mater.* **22**, 1453–1462 (2023).

4. R. Mundaca-Uribe, N. Askarinam, R. H. Fang, L. Zhang, J. Wang, Towards multifunctional robotic pills. *Nat. Biomed. Eng.* **8**, 1334–1346 (2024).

5. B. J. Nelson, S. Pané, Delivering drugs with microrobots. *Science* **382**, 1120–1122 (2023).

6. X. Yang, R. Tan, H. Lu, T. Fukuda, Y. Shen, Milli-scale cellular robots that can reconfigure morphologies and behaviors simultaneously. *Nat. Commun.* **13**, 4156 (2022).

7. B. Wang, K. F. Chan, K. Yuan, Q. Wang, X. Xia, L. Yang, H. Ko, Y. J. Wang, J. J. Y. Sung, P. W. Y. Chiu, L. Zhang, Endoscopy-assisted magnetic navigation of biohybrid soft microrobots with rapid endoluminal delivery and imaging. *Sci. Robot.* **6**, eabd2813 (2021).

8. W. Hu, G. Z. Lum, M. Mastrangeli, M. Sitti, Small-scale soft-bodied robot with multimodal locomotion. *Nature* **554**, 81–85 (2018).

9. R. Xie, Y. Cao, R. Sun, R. Wang, A. Morgan, J. Kim, S. J. P. Callens, K. Xie, J. Zou, J. Lin, K. Zhou, X. Lu, M. M. Stevens, Magnetically driven formation of 3D freestanding soft bioscaffolds. *Sci. Adv.* **10**, eadl1549 (2024).

10. S. K. Smoukov, S. Gangwal, M. Marquez, O. D. Velev, Reconfigurable responsive structures assembled from magnetic Janus particles. *Soft Matter* **5**, 1285–1292 (2009).

11. B. Bharti, O. D. Velev, Assembly of reconfigurable colloidal structures by multidirectional field-induced interactions. *Langmuir* **31**, 7897–7908 (2015).

12. J. Li, D. J. Mooney, Designing hydrogels for controlled drug delivery. *Nat. Rev. Mater.* **1**, 16071 (2016).

13. F. G. Downs, D. J. Lunn, M. J. Booth, J. B. Sauer, W. J. Ramsay, R. G. Klemperer, C. J. Hawker, H. Bayley, Multi-responsive hydrogel structures from patterned droplet networks. *Nat. Chem.* **12**, 363–371 (2020).

14. G. Hou, X. Zhang, F. Du, Y. Wu, X. Zhang, Z. Lei, W. Lu, F. Zhang, G. Yang, H. Wang, Z. Liu, R. Wang, Q. Ge, J. Chen, G. Meng, N. X. Fang, X. Qian, Self-regulated underwater phototaxis of a photoresponsive hydrogel-based phototactic vehicle. *Nat. Nanotechnol.* **19**, 77–84 (2024).

15. C. Ni, D. Chen, Y. Yin, X. Wen, X. Chen, C. Yang, G. Chen, Z. Sun, J. Wen, Y. Jiao, C. Wang, N. Wang, X. Kong, S. Deng, Y. Shen, R. Xiao, X. Jin, J. Li, X. Kong, Q. Zhao, T. Xie, Shape memory polymer with programmable recovery onset. *Nature* **622**, 748–753 (2023).

16. A. Pantula, B. Datta, Y. Shi, M. Wang, J. Liu, S. Deng, N. J. Cowan, T. D. Nguyen, D. H. Gracias, Untethered unidirectionally crawling gels driven by asymmetry in contact forces. *Sci. Robot.* **7**, eadd2903 (2022).

17. T. J. Ge, D. M. Roquero, G. H. Holton, K. E. Mach, K. Prado, H. Lau, K. Jensen, T. C. Chang, S. Conti, K. Sheth, S. X. Wang, J. C. Liao, A magnetic hydrogel for the efficient retrieval of kidney stone fragments during ureteroscopy. *Nat. Commun.* **14**, 3711 (2023).

18. Y. Zhao, C. Xuan, X. Qian, Y. Alsaid, M. Hua, L. Jin, X. He, Soft phototactic swimmer based on self-sustained hydrogel oscillator. *Sci. Robot.* **4**, eaax7112 (2019).

19. X. Q. Wang, G. W. Ho, Design of untethered soft material micromachine for life-like locomotion. *Mater. Today* **53**, 197–216 (2022).

20. A. Basu, L. B. Okello, N. Castellanos, S. Roh, O. D. Velev, Assembly and manipulation of responsive and flexible colloidal structures by magnetic and capillary interactions. *Soft Matter*

19, 2466–2485 (2023).

21. A. Basu, M. R. Clary, J. B. Tracy, C. K. Hall, O. D. Velev, Spatially confined assembly and immobilization of hierarchical nanoparticle architectures inside microdroplets in magnetic fields. *ACS Nano* **18**, 19814–19827 (2024).

22. H. H. Tran, Z. Xiang, M. J. Oh, Y. Liu, Z. Ren, C. Chen, N. Jaruchotiratanasakul, J. M. Kikkawa, D. Lee, H. Koo, E. Steager, Robotic microcapsule assemblies with adaptive mobility for targeted treatment of rugged biological microenvironments. *ACS Nano* **19**, 3265–3281 (2025).

23. X. Liu, N. Kent, A. Ceballos, R. Streubel, Y. Jiang, Y. Chai, P. Y. Kim, J. Forth, F. Hellman, S. Shi, D. Wang, B. A. Helms, P. D. Ashby, P. Fischer, T. P. Russell, Reconfigurable ferromagnetic liquid droplets. *Science* **365**, 264–267 (2019).

24. Y. Kim, X. Zhao, Magnetic soft materials and robots. *Chem. Rev.* **122**, 5317–5364 (2022).

25. Z. Xu, Z. Wu, M. Yuan, Y. Chen, W. Ge, Q. Xu, Versatile magnetic hydrogel soft capsule microrobots for targeted delivery. *iScience* **26**, 106727 (2023).

26. J. Yu, D. Jin, K. F. Chan, Q. Wang, K. Yuan, L. Zhang, Active generation and magnetic actuation of microrobotic swarms in bio-fluids. *Nat. Commun.* **10**, 5631 (2019).

27. Z. Chen, H. Wang, Y. Cao, Y. Chen, O. Akkus, H. Liu, C. Cao, Bio-inspired anisotropic hydrogels and their applications in soft actuators and robots. *Matter* **6**, 3803–3837 (2023).

28. Y. Kim, H. Yuk, R. Zhao, S. A. Chester, X. Zhao, Printing ferromagnetic domains for untethered fast-transforming soft materials. *Nature* **558**, 274–279 (2018).

29. J. Zhang, Z. Ren, W. Hu, R. H. Soon, I. C. Yasa, Z. Liu, M. Sitti, Voxelated three-dimensional miniature magnetic soft machines via multimaterial heterogeneous assembly. *Sci. Robot.* **6**, eabf0112 (2021).

30. Y. Kim, G. A. Parada, S. Liu, X. Zhao, Ferromagnetic soft continuum robots. *Sci. Robot.* **4**,

eaax7329 (2019).

31. Y. Kim, E. Genevriere, P. Harker, J. Choe, M. Balicki, R. W. Regenhardt, J. E. Vranic, A. A. Dmytriw, A. B. Patel, X. Zhao, Telerobotic neurovascular interventions with magnetic manipulation. *Sci. Robot.* **7**, eabg9907 (2022).

32. J. Yan, M. Bloom, S. C. Bae, E. Luijten, S. Granick, Linking synchronization to self-assembly using magnetic Janus colloids. *Nature* **491**, 578–581 (2012).

33. Q. Peng, S. Wang, J. Han, C. Huang, H. Yu, D. Li, M. Qiu, S. Cheng, C. Wu, M. Cai, S. Fu, B. Chen, X. Wu, S. Du, T. Xu, Thermal and magnetic dual-responsive catheter-assisted shape memory microrobots for multistage vascular embolization. *Research* **7**, 0339 (2024).

34. L. Yang, J. L. Jiang, X. J. Gao, Q. L. Wang, Q. Dou, L. Zhang, Autonomous environment-adaptive microrobot swarm navigation enabled by deep learning-based real-time distribution planning. *Nat. Mach. Intell.* **4**, 480–493 (2022).

35. J. Yu, B. Wang, X. Du, Q. Wang, L. Zhang, Ultra-extensible ribbon-like magnetic microswarm. *Nat. Commun.* **9**, 3260 (2018).

36. G. R. Lichtenstein, M. T. Abreu, R. Cohen, W. Tremaine, American Gastroenterological Association, American Gastroenterological Association Institute technical review on corticosteroids, immunomodulators, and infliximab in inflammatory bowel disease. *Gastroenterology* **130**, 940–987 (2006).

37. E. Minaei, M. Ranson, M. Aghmesheh, R. Sluyter, K. L. Vine, Enhancing pancreatic cancer immunotherapy: Leveraging localized delivery strategies through the use of implantable devices and scaffolds. *J. Control. Release* **373**, 145–160 (2024).

38. J. Li, S. Thamphiwatana, W. Liu, B. Esteban-Fernandez de Avila, P. Angsantikul, E. Sandraz, J. Wang, T. Xu, F. Soto, V. Ramez, X. Wang, W. Gao, L. Zhang, J. Wang, Enteric micromotor can selectively position and spontaneously propel in the gastrointestinal tract. *ACS Nano* **10**, 9536–9542 (2016).

39. M. P. Kummer, J. J. Abbott, B. E. Kratochvil, R. Borer, A. Sengul, B. J. Nelson, OctoMag: An electromagnetic system for 5-DOF wireless micromanipulation. *IEEE Trans. Robot.* **26**, 1006–1017 (2010).

40. R. Dreyfus, Q. Boehler, S. Lyttle, P. Gruber, J. Lussi, C. Chautems, S. Gervasoni, J. Berberat, D. Seibold, N. Ochsenbein-Kölble, M. Reinehr, M. Weisskopf, L. Remonda, B. J. Nelson, Dexterous helical magnetic robot for improved endovascular access. *Sci. Robot.* **9**, eadh0298 (2024).

41. S. A. Abbasi, A. Ahmed, S. Noh, N. L. Gharamaleki, S. Kim, A. M. M. B. Chowdhury, J.-y. Kim, S. Pané, B. J. Nelson, H. Choi, Autonomous 3D positional control of a magnetic microrobot using reinforcement learning. *Nat. Mach. Intell.* **6**, 92–105 (2024).

42. L. Yang, J. Jiang, F. Ji, Y. Li, K.-L. Yung, A. Ferreira, L. Zhang, Machine learning for micro- and nanorobots. *Nat. Mach. Intell.* **6**, 605–618 (2024).

43. G. Go, A. Yoo, K. T. Nguyen, M. Nan, B. A. Darmawan, S. Zheng, B. Kang, C.-S. Kim, D. Bang, S. Lee, K.-P. Kim, S. S. Kang, K. M. Shim, S. E. Kim, S. Bang, D.-H. Kim, J.-O. Park, E. Choi, Multifunctional microrobot with real-time visualization and magnetic resonance imaging for chemoembolization therapy of liver cancer. *Sci. Adv.* **8**, eabq8545 (2022).

44. H. Han, X. Ma, W. Deng, J. Zhang, S. Tang, O. S. Pak, L. Zhu, E. Criado-Hidalgo, C. Gong, E. Karshalev, J. Yoo, M. You, A. Liu, C. Wang, H. K. Shen, P. N. Patel, C. L. Hays, P. J. Gunnarson, L. Li, Y. Zhang, J. O. Dabiri, L. V. Wang, M. G. Shapiro, D. Wu, Q. Zhou, J. R. Greer, W. Gao, Imaging-guided bioresorbable acoustic hydrogel microrobots. *Sci. Robot.* **9**, eadp3593 (2024).

45. S. Jeon, S. Kim, S. Ha, S. Lee, E. Kim, S. Y. Kim, S. H. Park, J. H. Jeon, S. W. Kim, C. Moon, B. J. Nelson, J. Y. Kim, S. W. Yu, H. Choi, Magnetically actuated microrobots as a platform for stem cell transplantation. *Sci. Robot.* **4**, (2019).

46. Y. Cao, J. Tan, H. Zhao, T. Deng, Y. Hu, J. Zeng, J. Li, Y. Cheng, J. Tang, Z. Hu, K. Hu, B. Xu, Z. Wang, Y. Wu, P. E. Lobie, S. Ma, Bead-jet printing enabled sparse mesenchymal stem cell patterning augments skeletal muscle and hair follicle regeneration. *Nat. Commun.* **13**, 7463

(2022).

47. J. A. Anderson, J. Glaser, S. C. Glotzer, HOOMD-blue: A Python package for high-performance molecular dynamics and hard particle Monte Carlo simulations. *Comput. Mater. Sci.* **173**, 109363 (2020).

48. V. Ramasubramani, B. D. Dice, E. S. Harper, M. P. Spellings, J. A. Anderson, S. C. Glotzer, freud: A software suite for high throughput analysis of particle simulation data. *Comput. Phys. Commun.* **254**, 107275 (2020).

49. C. S. Adorf, P. M. Dodd, V. Ramasubramani, S. C. Glotzer, Simple data and workflow management with the signac framework. *Comput. Mater. Sci.* **146**, 220–229 (2018).

50. Q. Ze, S. Wu, J. Dai, S. Leanza, G. Ikeda, P. C. Yang, G. Iaccarino, R. R. Zhao, Spinning-enabled wireless amphibious origami millirobot. *Nat. Commun.* **13**, 3118 (2022).

51. E. E. Niedert, C. Bi, G. Adam, E. Lambert, L. Solorio, C. J. Goergen, D. J. Cappelleri, A tumbling magnetic microrobot system for biomedical applications. *Micromachines* **11**, 861 (2020).

52. C. Li, G. C. Lau, H. Yuan, A. Aggarwal, V. L. Dominguez, S. Liu, H. Sai, L. C. Palmer, N. A. Sather, T. J. Pearson, D. E. Freedman, P. K. Amiri, M. O. de la Cruz, S. I. Stupp, Fast and programmable locomotion of hydrogel-metal hybrids under light and magnetic fields. *Sci. Robot.* **5**, eabb9822 (2020).

53. S. Yu, T. Li, F. Ji, S. Zhao, K. Liu, Z. Zhang, W. Zhang, Y. Mei, Trimer-like microrobots with multimodal locomotion and reconfigurable capabilities. *Mater. Today Adv.* **14**, 100231 (2022).

54. Y. W. Ju, R. Hu, Y. Xie, J. P. Yao, X. X. Li, Y. L. Lv, X. T. Han, Q. L. Cao, L. Li, Reconfigurable magnetic soft robots with multimodal locomotion. *Nano Energy* **87**, 106169 (2021).

55. Y. Wu, X. Dong, J. K. Kim, C. Wang, M. Sitti, Wireless soft millirobots for climbing three-dimensional surfaces in confined spaces. *Sci. Adv.* **8**, eabn3431 (2022).

56. X. Hui, J. Luo, R. Wang, H. Sun, Multiresponsive microactuator for ultrafast submillimeter robots. *ACS Nano* **17**, 6589–6600 (2023).

57. J. J. Wie, M. R. Shankar, T. J. White, Photomotility of polymers. *Nat. Commun.* **7**, 13260 (2016).

58. J. X. Wu, W. F. Ai, K. Hou, C. F. Zhang, Y. Long, K. Song, Light-driven soft climbing robot based on negative pressure adsorption. *Chem. Eng. J.* **466**, 143131 (2023).

59. Y. Wu, J. K. Yim, J. Liang, Z. Shao, M. Qi, J. Zhong, Z. Luo, X. Yan, M. Zhang, X. Wang, R. S. Fearing, R. J. Full, L. Lin, Insect-scale fast moving and ultrarobust soft robot. *Sci. Robot.* **4**, eaax1594 (2019).

60. G. Gu, J. Zou, R. Zhao, X. Zhao, X. Zhu, Soft wall-climbing robots. *Sci. Robot.* **3**, eaat2874 (2018).

61. W. Haomachai, D. H. Shao, W. Wang, A. H. Ji, Z. D. Dai, P. Manoonpong, Lateral undulation of the bendable body of a Gecko-inspired robot for energy-efficient inclined surface climbing. *IEEE Robot. Autom. Lett.* **6**, 7917–7924 (2021).

62. Y. F. Chen, N. Doshi, B. Goldberg, H. Q. Wang, R. J. Wood, Controllable water surface to underwater transition through electrowetting in a hybrid terrestrial-aquatic microrobot. *Nat. Commun.* **9**, 2495 (2018).

63. S. D. de Rivaz, B. Goldberg, N. Doshi, K. Jayaram, J. Zhou, R. J. Wood, Inverted and vertical climbing of a quadrupedal microrobot using electroadhesion. *Sci. Robot.* **3**, eaau3038 (2018).

64. W. B. Pang, S. W. Xu, J. Wu, R. H. Bo, T. Q. Jin, Y. Xiao, Z. Liu, F. Zhang, X. Cheng, K. Bai, H. L. Song, Z. G. Xue, L. Wen, Y. H. Zhang, A soft microrobot with highly deformable 3D actuators for climbing and transitioning complex surfaces. *Proc. Natl. Acad. Sci. U.S.A.* **119**, e2215028119 (2022).

65. Y. Q. Fu, H. Q. Wang, Y. L. Zi, X. Q. Liang, A multifunctional robotic system toward moveable sensing and energy harvesting. *Nano Energy* **89**, 106368 (2021).

66. Y. F. Chen, N. Doshi, R. J. Wood, Inverted and inclined climbing using capillary adhesion in a quadrupedal insect-scale robot. *IEEE Robot. Autom. Lett.* **5**, 4820–4827 (2020).

67. Y. Tang, Y. Chi, J. Sun, T. H. Huang, O. H. Maghsoudi, A. Spence, J. Zhao, H. Su, J. Yin, Leveraging elastic instabilities for amplified performance: Spine-inspired high-speed and high-force soft robots. *Sci. Adv.* **6**, eaaz6912 (2020).

68. X. Yang, L. Chang, N. O. Pérez-Arancibia, An 88-milligram insect-scale autonomous crawling robot driven by a catalytic artificial muscle. *Sci. Robot.* **5**, eaba0015 (2020).

69. F. Zhai, Y. Y. Feng, Z. Y. Li, Y. X. Xie, J. Ge, H. Wang, W. Qiu, W. Feng, 4D-printed untethered self-propelling soft robot with tactile perception: Rolling, racing, and exploring. *Matter* **4**, 3313–3326 (2021).

70. A. van Reenen, A. M. de Jong, J. M. den Toonder, M. W. Prins, Integrated lab-on-chip biosensing systems based on magnetic particle actuation – A comprehensive review. *Lab Chip* **14**, 1966–1986 (2014).
